# Two new insulator proteins, Pita and ZIPIC, target CP190 to chromatin

**DOI:** 10.1101/gr.174169.114

**Published:** 2015-01

**Authors:** Oksana Maksimenko, Marek Bartkuhn, Viacheslav Stakhov, Martin Herold, Nickolay Zolotarev, Theresa Jox, Melanie K. Buxa, Ramona Kirsch, Artem Bonchuk, Anna Fedotova, Olga Kyrchanova, Rainer Renkawitz, Pavel Georgiev

**Affiliations:** 1 Laboratory of Gene Expression Regulation in Development, Institute of Gene Biology, Russian Academy of Sciences, Moscow 119334, Russia;; 2 Institute for Genetics, Justus-Liebig-University Giessen, Heinrich-Buff-Ring, D-35392 Giessen, Germany;; 3 Group of Transcriptional Regulation, Institute of Gene Biology, Russian Academy of Sciences, Moscow 119334, Russia;; 4 Department of the Control of Genetic Processes, Institute of Gene Biology, Russian Academy of Sciences, Moscow 119334, Russia

## Abstract

Insulators are multiprotein–DNA complexes that regulate the nuclear architecture. The *Drosophila* CP190 protein is a cofactor for the DNA-binding insulator proteins Su(Hw), CTCF, and BEAF-32. The fact that CP190 has been found at genomic sites devoid of either of the known insulator factors has until now been unexplained. We have identified two DNA-binding zinc-finger proteins, Pita, and a new factor named ZIPIC, that interact with CP190 in vivo and in vitro at specific interaction domains. Genomic binding sites for these proteins are clustered with CP190 as well as with CTCF and BEAF-32. Model binding sites for Pita or ZIPIC demonstrate a partial enhancer-blocking activity and protect gene expression from PRE-mediated silencing. The function of the CTCF-bound *MCP* insulator sequence requires binding of Pita. These results identify two new insulator proteins and emphasize the unifying function of CP190, which can be recruited by many DNA-binding insulator proteins.

Insulators in the *Drosophila* and vertebrate genomes have been identified based on their ability to disrupt the communication between an enhancer and a promoter when inserted between them (Raab and Kamakaka 2010; Ghirlando et al. 2012; Herold et al. 2012; Matzat and Lei 2013; Chetverina et al. 2014; Kyrchanova and Georgiev 2014). The growing amount of data show that insulator proteins fulfil an architectural function in mediating inter- and intrachromosomal interactions and in contacting regulatory elements such as promoters or enhancers (Maksimenko and Georgiev 2014).

The best studied *Drosophila* insulator proteins, dCTCF (homolog of vertebrate insulator protein CTCF) and Su(Hw) are DNA-binding zinc-finger proteins (Herold et al. 2012; Matzat and Lei 2013; Kyrchanova and Georgiev 2014). Binding sites for dCTCF have been identified in the insulators that separate functional regulatory domains of the *bithorax* complex and in many promoter regions (Moon et al. 2005; Holohan et al. 2007; Mohan et al. 2007; Nègre et al. 2010, 2011; Ni et al. 2012). The Su(Hw) protein more frequently associates with intergenic sites (Adryan et al. 2007; Bushey et al. 2009; Nègre et al. 2010, 2011; Soshnev et al. 2012, 2013). As shown in a transgenic assay, dCTCF and Su(Hw) binding sites can support specific distant interactions (Kyrchanova et al. 2008a,b), which suggests a key role for these proteins in organizing chromatin architecture.

The Su(Hw), dCTCF, and BEAF-32 proteins interact with Centrosomal Protein 190 kD, named CP190 (Pai et al. 2004; Gerasimova et al. 2007; Mohan et al. 2007; Bartkuhn et al. 2009; Oliver et al. 2010; Liang et al. 2014). CP190 (1096 amino acids) contains an N-terminal BTB/POZ domain, an aspartic-acid-rich D-region, four C2H2 zinc-finger motifs, and a C-terminal E-rich domain (Oliver et al. 2010; Ahanger et al. 2013). The BTB domain of CP190 forms stable homodimers that may be involved in protein–protein interactions (Oliver et al. 2010; Bonchuk et al. 2011). In addition to these motifs, CP190 also contains a centrosomal targeting domain (M) responsible for its localization to centrosomes during mitosis (Butcher et al. 2004). It has been shown that CP190 is recruited to chromatin via its interaction with the Su(Hw) and dCTCF proteins (Pai et al. 2004; Mohan et al. 2007). Inactivation of CP190 affects the activity of the dCTCF-dependent insulator *Fab-8* from the *bithorax* complex (Gerasimova et al. 2007; Mohan et al. 2007; Moshkovich et al. 2011) and the *gypsy* insulator, which contains 12 binding sites for the Su(Hw) protein (Pai et al. 2004). Binding of Su(Hw) and CP190 at *gypsy*-like sites is mutually dependent, indicating a stabilizing role of CP190 in these cases (Schwartz et al. 2012).

Recent genome-wide ChIP-chip studies provide evidence for an extensive overlap of the CP190 distribution pattern with dCTCF, BEAF-32, and Su(Hw) insulator proteins and the promoters of active genes (Bartkuhn et al. 2009; Bushey et al. 2009; Nègre et al. 2010, 2011; Schwartz et al. 2012; Soshnev et al. 2012). Very recently, it has been demonstrated that CP190 bridges DNA-bound insulator factors with promoters (Liang et al. 2014). These data support the model that CP190 has a global role in the function of insulator proteins. However, there are a number of sites in the *Drosophila* genome where CP190 does not colocalize with any known insulator DNA binding protein (IBP), suggesting that there may be some other proteins that recruit CP190 to chromatin (Schwartz et al. 2012).

To identify new factors that associate with CP190, we purified the FLAG-tagged CP190 protein from S2 cells and identified two zinc-finger proteins, CG7928 and Pita, which were shown to interact with CP190 in vivo and in vitro. Genome-wide identification of binding sites for Pita and CG7928 in S2 cells revealed their extensive colocalization with CP190, providing evidence for direct interactions between these proteins, which was supported by binding and in vivo functional assays. Based on these results we termed CG7928 the “zinc-finger protein interacting with CP190” (ZIPIC).

## Results

### Identification of Pita and ZIPIC as partners of the CP190 protein

**Figure 1.**
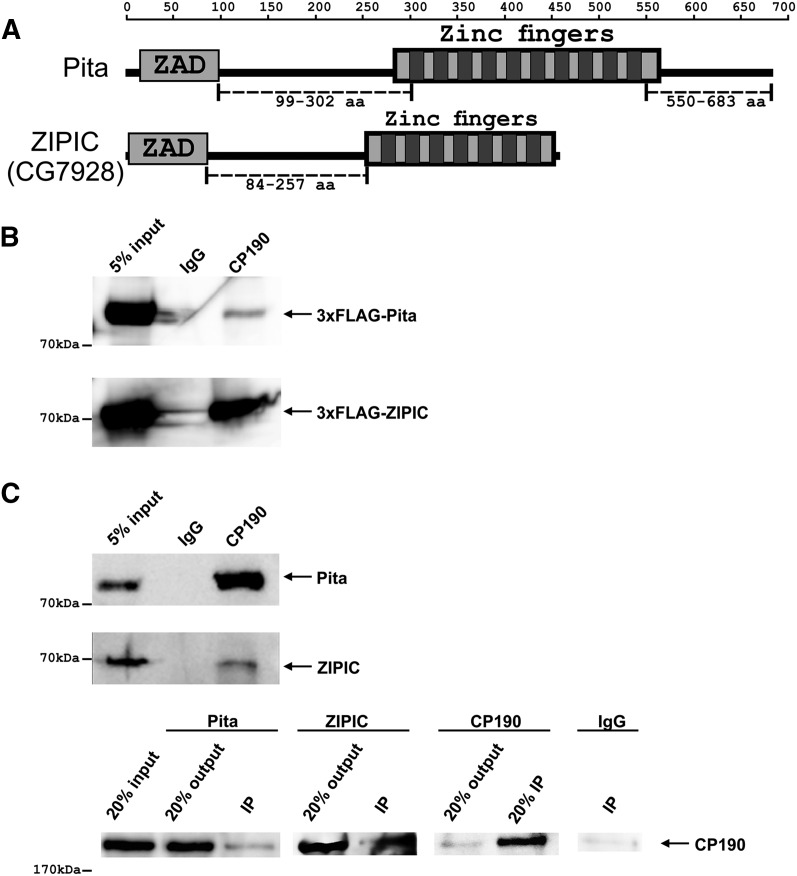
Interaction of CP190 with Pita and ZIPIC proteins. (*A*) Structure of full-length Pita and ZIPIC proteins containing the ZAD domain and C2H2-type zinc fingers. The scale shows the number of amino acid residues. Broken lines indicate regions used to prepare antibodies. (*B*) Nuclear extracts from *Drosophila* S2 cells cotransfected with CP190 and 3 × FLAG-Pita/ ZIPIC were immunoprecipitated with antibodies against CP190 (using nonspecific IgG as a negative control), and the immunoprecipitates were analyzed by Western blotting for the presence of FLAG-tagged proteins. (*C*) Nuclear extracts from *Drosophila* embryos were immunoprecipitated with antibodies against CP190, Pita, or ZIPIC (using nonspecific IgG as a negative control), and the immunoprecipitates (IP) were analyzed by Western blotting for the presence of Pita, ZIPIC, and CP190. Inputs show the starting samples of nuclear extract; outputs are supernatant after sedimentation of immunoprecipitated material.

CP190 plays a central role in *Drosophila* boundary and domain formation (for review, see Ahanger et al. 2013) To identify unknown DNA binding factors that might be associated with a chromatin domain function of CP190, we searched for zinc-finger proteins that can interact with CP190. We purified CP190 from an extract prepared from S2 cells stably expressing a tagged FLAG-*CP190* transgene. Purified material from extracts with or without CuSO_4_ induction was analyzed using mass spectrometry. Proteins were considered to be interacting when they were enriched more than threefold in the induced sample compared to the uninduced material. This resulted in the identification of several known CP190 interactors, as well as of factors known to be enriched at domain boundaries: These were Map60 (CP60) (Kellogg et al. 1995), CTCF (Gerasimova et al. 2007; Mohan et al. 2007), Su(Hw) (Gdula and Corces 1997), Mod(mdg4) (Pai et al. 2004), Ibf1 and Ibf2 (Cuartero et al. 2014), Chromator (Sexton et al. 2012; Van Bortle et al. 2012), and Putzig (Z4) (Cuartero et al. 2014). In addition to these known factors, we found the two zinc-finger proteins, Pita and ZIPIC (Fig. 1A), which we chose for further analysis.

Pita or ZIPIC interaction with CP190 was confirmed by coimmunoprecipitation of CP190 and 3×FLAG-tagged Pita or ZIPIC transfected S2 cells (Fig. 1B). To further examine the new proteins, we prepared polyclonal antibodies against Pita (99–302 and 550–683aa regions) and ZIPIC (84–257aa region). The specificities of these antibodies were confirmed by RNAi knockdown of the corresponding protein in S2 cells (Supplemental Fig. S1A). Coimmunoprecipitation of CP190 with Pita or ZIPIC in the embryonic extract provided evidence for localization of these proteins in the same protein complexes in vivo (Fig. 1C). We also observed a weak interaction between Pita and ZIPIC in coimmunoprecipitation from embryonic extract (Supplemental Fig. S1B). To corroborate this finding, we tested whether CP190, Pita, and ZIPIC colocalize on polytene chromosomes of third-instar larvae (Supplemental Fig. S1C). CP190 was detected at almost all Pita and ZIPIC sites, which is indicative of its interaction with either of these proteins on the polytene chromosomes.

Taken together these results suggest that Pita and ZIPIC are interaction partners of the CP190 protein.

### Mapping the domains responsible for interactions between insulator proteins

**Figure 2.**
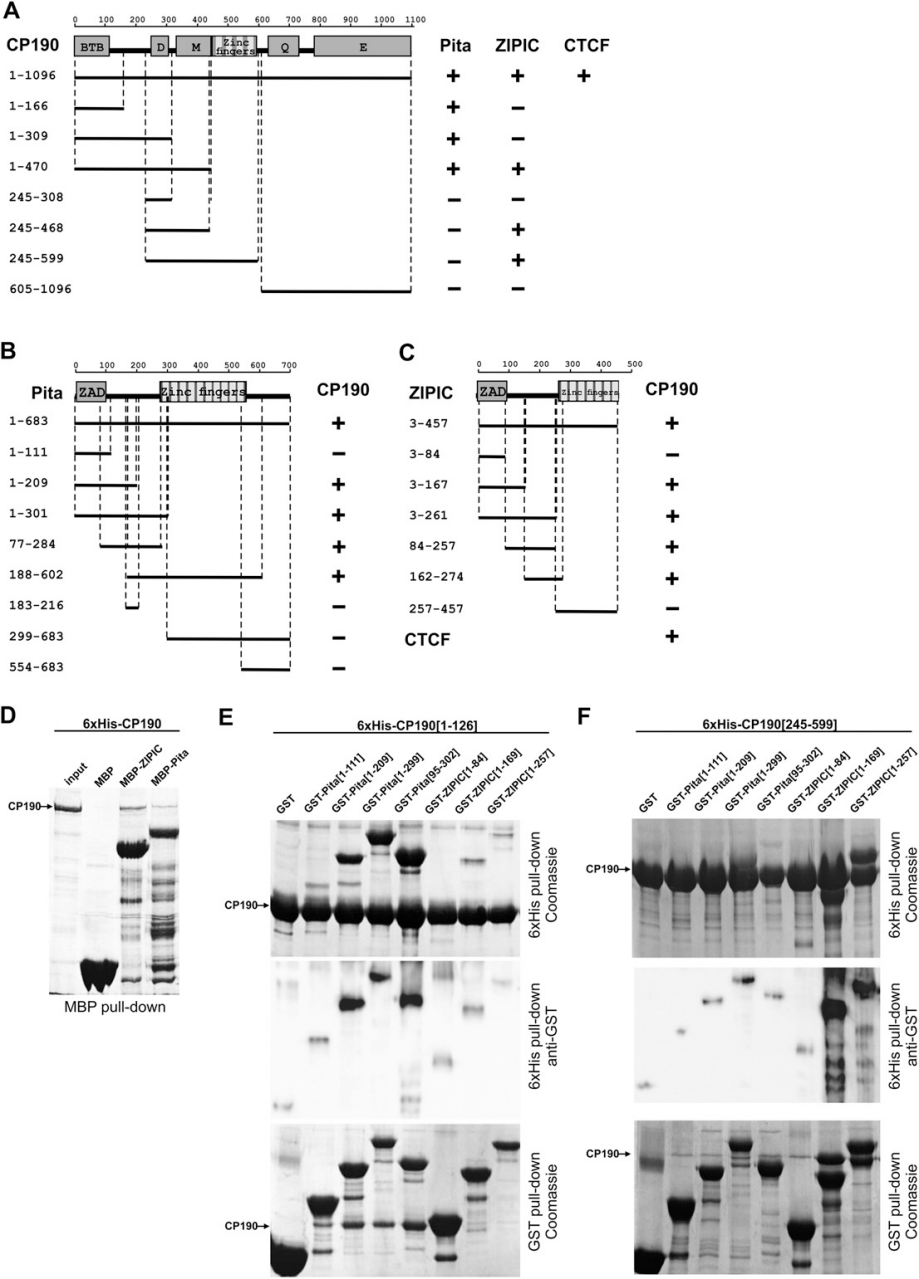
Identification of interacting domains of CP190, Pita, and ZIPIC proteins. (*A*) Localization of CP190 domains interacting with Pita and ZIPIC in yeast two-hybrid assay. In the structural scheme of full-length CP190, protein domains are shown as boxes, and lines indicate the different deletion fragments. The horizontal scale and figures on the *left* show the positions of amino acid residues. Different fragments of CP190 were fused to the GAL4 DNA-binding domain and tested for interaction with Pita and ZIPIC fused to the GAL4 activating domain. All CP190 fragments were tested for the absence of interaction with the GAL4 activating domain alone, whereas Pita and ZIPIC were tested for the absence of interaction with GAL4 DNA-binding domain alone. The results are summarized in columns on the *right*, with the “+” and “−” signs referring to a strong interaction or the absence of interaction, respectively. Interaction of CP190 with dCTCF was used as a positive control. (*B*) Localization of Pita domains interacting with CP190 in a yeast two-hybrid assay. Different fragments of Pita were fused to the GAL4 activating domain and tested for interaction with CP190 fused to the GAL4 DNA-binding domain. All Pita fragments were tested for the absence of interaction with the GAL4 DNA-binding domain alone. Other designations are the same as in *A*. (*C*) Localization of ZIPIC domains interacting with CP190 in a yeast two-hybrid assay. Different fragments of CG7928 were fused to the GAL4 activating domain and tested for interaction with CP190 fused to the GAL4 DNA-binding domain. All ZIPIC fragments were tested for the absence of interaction with the GAL4 DNA-binding domain alone. Other designations are the same as in *A*. (*D*) Interaction of full-length recombinant CP190 and Pita/ ZIPIC in MBP pull-down assay. Beads with bound MBP-Pita, MBP-ZIPIC, or MBP alone were incubated with 6 × His-CP190, and the precipitated proteins were resolved by SDS-PAGE and stained with Coomassie. (*E*,*F*) Interaction of CP190 fragments [1–126] (*E*) and [245–599] (*F*) with different fragments of Pita and ZIPIC in GST and 6 × His pull-down assays. The positions of amino acids are indicated by square brackets. In the 6 × His pull-down assay, beads with bound 6 × His-CP190 were incubated with each GST-tagged Pita or ZIPIC fragment or GST alone. In the GST pull-down assay, beads carrying GST-tagged Pita or ZIPIC fragment or GST alone were incubated with 6 × His-CP190 fragments. The precipitated proteins were resolved by SDS-PAGE and stained with Coomassie. Additionally, the precipitated proteins were immunoblotted with anti-GST antibodies in the case of the 6 × His pull-down assay.

To determine the domains involved in the interaction of CP190 with Pita and ZIPIC, we carried out yeast two-hybrid and in vitro pull-down assays. The yeast two-hybrid assay confirmed the interaction between CP190 and Pita or ZIPIC (Fig. 2A) and allowed us to narrow down the interaction region in CP190. Pita interacted with the BTB domain, whereas ZIPIC interacted with the region overlapping the M domain. The yeast two-hybrid assay was also used to identify the CP190-interacting domains of Pita and ZIPIC (Fig. 2B,C). Pita contains 10 zinc fingers at the carboxy (C) terminus and ZIPIC seven. Both have a zinc-finger associated domain (ZAD) at the amino (N) terminus (Fig. 2B,C). The ZAD domain characterizes the single largest subfamily of zinc-finger genes in *Drosophila* and may be involved in the coordination of zinc ion binding (Chung et al. 2002). We tested different fragments of the Pita protein and found that CP190 interacted with at least two sites in its 95–301aa region between the ZAD and zinc-finger domains (Fig. 2B; Supplemental Fig. S2). ZIPIC interacted with its 84–257aa region with CP190 (Fig. 2C; Supplemental Fig. S2). Very likely, CP190 interacts with two subregions of ZIPIC as both fragments, 3–167aa and 162–274aa, are positive for interaction.

The MBP pull-down assay also confirmed the interaction of Pita and ZIPIC with CP190 (Fig. 2D). We then used 6 × His and GST pull-down assays to examine the interaction between the 6 × His-tagged BTB domain (1–126aa) or the M domain (245–599aa) of CP190 and GST-tagged fragments of Pita or ZIPIC. The results confirmed that the BTB domain of CP190 interacted with the Pita^95–209^ fragment (Fig. 2E), and the CP190 M domain indeed interacted with the ZIPIC^84–257^ fragment (Fig. 2F).

### Pita and ZIPIC binding sites are strongly correlated with promoter regions and CP190 binding sites

**Figure 3.**
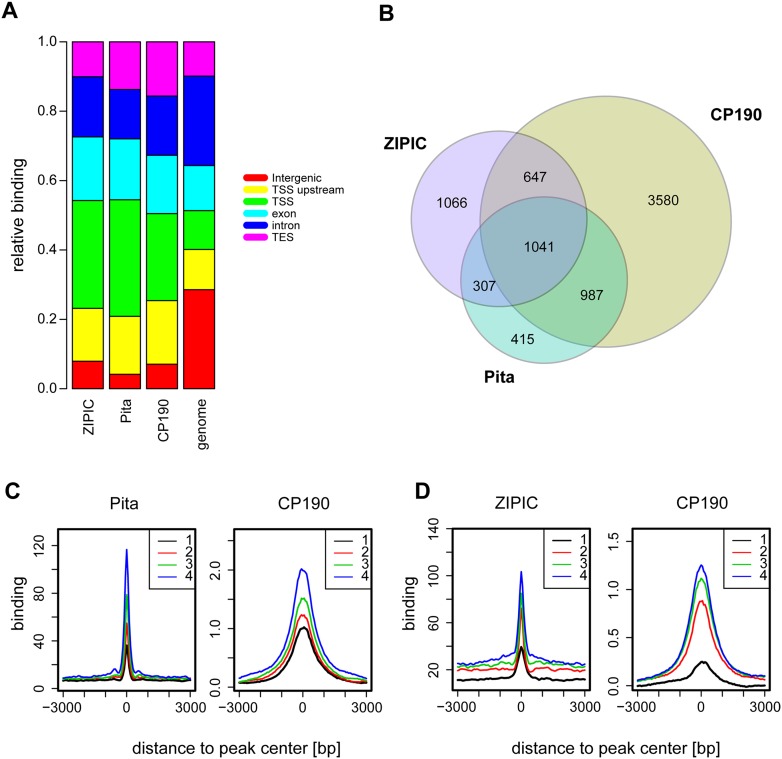
Colocalization of CP190 with Pita or ZIPIC. (*A*) Distribution of genomic elements across regions of significant Pita, ZIPIC, and CP190 binding compared to their background distribution in the genome: ([green] TSS; [yellow] TSS upstream; [light blue] exon; [dark blue] intron; [purple] TES; [red] intergenic sites). (*B*) Venn diagram of the overlap between Pita, ZIPIC, and CP190 peaks. (*C*,*D*) Pita or ZIPIC binding regions were divided into four groups with respect to binding strength, from the lowest (1) to highest (4), and the average profiles for Pita (*C*) and ZIPIC (*D*) were plotted next to the average CP190 signals across the same sites.

In order to identify the binding sites for Pita and ZIPIC in the *Drosophila* genome, we performed chromatin immunoprecipitation experiments with subsequent sequencing using Illumina’s massive parallel sequencing technology. Optical inspection of coverage vectors revealed the presence of many narrow peaks, which often coincided with gene promoters (Supplemental Fig. S3). Comparing specific ChIP patterns to sequenced input DNA resulted in the identification of a total of 2750 peaks for Pita and 3061 peaks for ZIPIC. When we correlated the distribution of these peaks with genomic annotations, we found that they were indeed concentrated in promoter regions and were relatively rare in intergenic and intronic regions (Fig. 3A).

In order to estimate to what extent the Pita and ZIPIC peaks overlap with known CP190 binding sites, we analyzed ChIP-chip data from the modENCODE Consortium (The modENCODE Consortium et al. 2010). As expected, we found that most of these peaks (74% for Pita and 55% for ZIPIC) overlapped with a CP190 binding site, whereas 57% of CP190 peaks did not show any overlap with either ZIPIC or Pita peaks (Fig. 3B). Therefore, only a part of CP190 binding events can be explained by recruitment through Pita or ZIPIC. A high proportion of Pita and ZIPIC sites overlap with each other and, in many instances, also with CP190 sites (Fig. 3B). To test whether the binding strength of both factors is correlated with that of CP190, we grouped all of these sites into four groups with respect to binding strength, from the lowest (1) to highest (4). We then plotted the corresponding average profiles for Pita (Fig. 3C) and for ZIPIC (Fig. 3D) next to the average CP190 signals across the same sites. This analysis demonstrates that CP190 binding is proportional to that of Pita and ZIPIC binding. From this it can be concluded that binding of adjacent DNA binding factors may be cooperatively increased, and that this is further stabilized by CP190 binding via multiple contacts to Pita and to ZIPIC as well as to other DNA-bound insulator factors in the vicinity (see below).

### Identification of associated binding motifs

**Figure 4.**
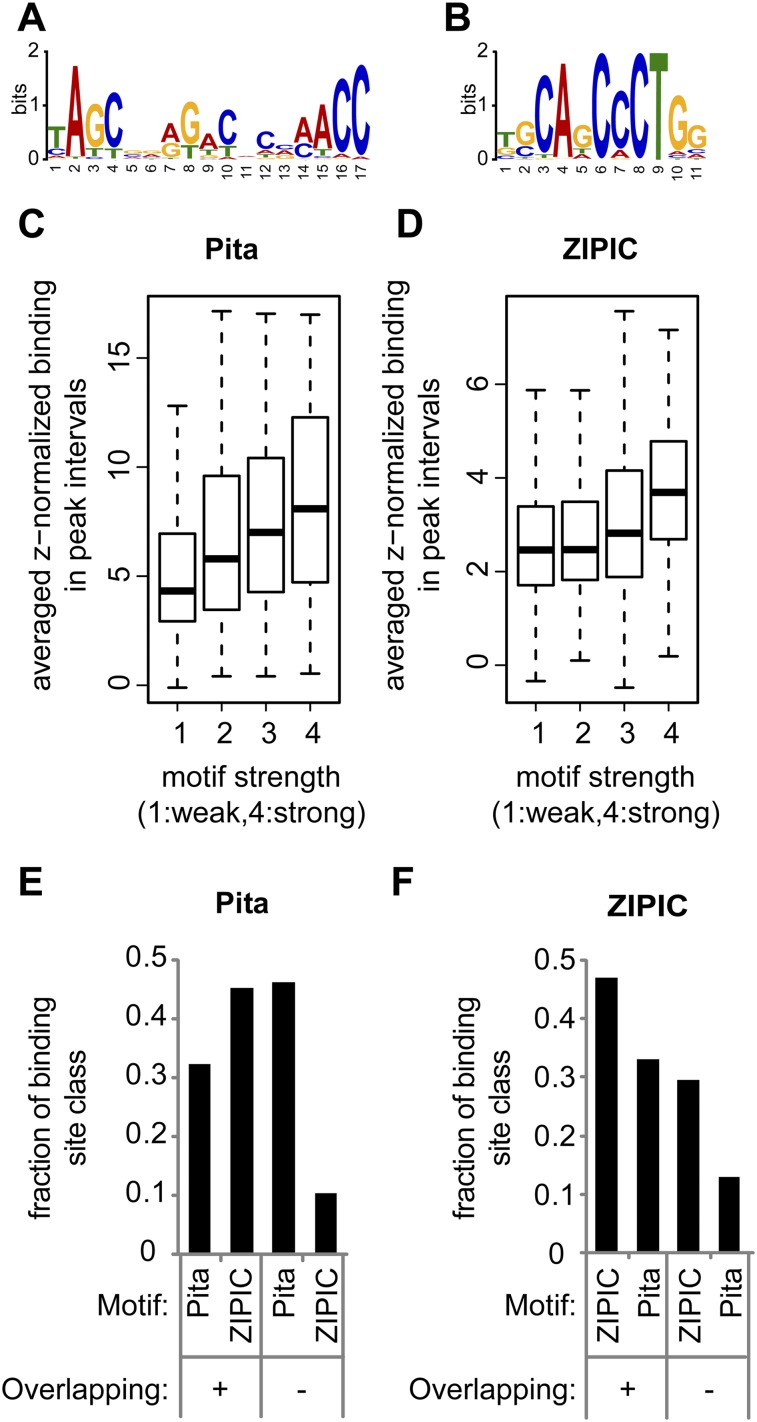
Motif specificity for Pita and ZIPIC. (*A*,*B*) The top motifs identified within (*A*) Pita and (*B*) ZIPIC peaks as a sequence logo. (*C*,*D*) The binding of (*C*) Pita and (*D*) ZIPIC is correlated with motif conservation. Peaks were classified with respect to similarity to the best matching consensus-like sequence and grouped accordingly: from (1) weak similarity to (4) high similarity. (*E*,*F*) Peaks of (*E*) Pita and (*F*) ZIPIC were divided into two groups by the criterion of overlap with peaks of the other protein (overlapping versus nonoverlapping peaks), and both groups were analyzed for the presence of ZIPIC and Pita motifs.

Since Pita and ZIPIC contain multiple zinc fingers, we suggested that many of the detected ChIP-seq signals were a result of direct DNA-binding events. To identify potential sequence motifs where such events could indeed take place, we performed a de novo motif search using the MEME program (Machanick and Bailey 2011), concentrating on a ± 20-bp region around the peak maxima of Pita and ZIPIC. For Pita, we were able to identify a 17-mer sequence with an *E*-value of 3.9 × 10^−187^ (Fig. 4A). It should be noted that we did not find related motifs in the TRANSFAC or JASPAR databases; however, the preceding sequence is very similar to a sequence recently identified by Schwartz et al. (2012) as a CP190-binding motif devoid of the insulator factors dCTCF, BEAF-32, TRL (also known as GAF), or Su(Hw). For ZIPIC, we identified an 11-mer with an *E*-value of 3 × 10^−224^ (Fig. 4B), which also had no counterparts in the aforementioned databases. We constructed position specific scoring matrices and scanned the complete genome for the occurrence of these two motifs. Under the specified thresholds, we identified 1090 Pita peaks and 1147 ZIPIC peaks that overlapped the respective motifs.

To estimate the significance of the observed overlap, we performed simulations with randomized peak sets (1000 iterations) and found that there was not a single situation as extreme as in the observed case (*P* < 0.001, data not shown). In addition, we analyzed the dependence of the average binding strength across peak intervals on the conservation level of the associated motif. For this purpose, we calculated the best matching motif score for each peak and grouped the peaks according to these scores into four classes, from (1) low to (4) high similarity. When we plotted the binding strength as a function of these classes, we found that the binding strength correlated with the level of similarity between the observed motif and the identified consensus (Figs. 4C,D). Taken together, these data strongly suggest that the binding events detected by ChIP-seq are in many instances dependent on direct DNA-interactions of Pita and ZIPIC with the identified motifs.

A large degree of overlap between ZIPIC and Pita binding, as noted above, might be caused by one of the two factors binding to the other one. Alternatively, both factors could bind to adjacent DNA sequences with their respective consensus specificity. To distinguish between these alternatives, we grouped Pita and ZIPIC binding peaks into overlapping and nonoverlapping cases. As expected for the nonoverlapping peaks, the Pita-specific consensus sequence was found within the Pita peaks, and the ZIPIC-specific consensus was enriched in the ZIPIC peaks (Fig. 4E,F). In the case of the overlapping peaks, both *consensi* were found with a similar frequency. These data indicate that, although Pita and ZIPIC binding are often colocalized in the genome, their sequence specific binding is mediated through their respective binding motif.

To test for interdependence of CP190 and ZIPIC, or of CP190 and Pita binding to chromatin, we selected three binding regions for Pita (60A9L, 100B7, 100C) and four binding regions for ZIPIC (57B5, 60A9R, 66E5, 67B6) (Supplemental Fig. S4), all of them containing consensus binding sites for either protein. The binding of Pita or ZIPIC to the selected regions was confirmed by EMSA (Supplemental Figs. S5A,B). Using chromatin immunoprecipitation, we also confirmed the in vivo binding of CP190 and Pita to the Pita-binding regions (Supplemental Fig. S5A) and of CP190 and ZIPIC to the ZIPIC-binding regions (Supplemental Fig. S5B) in S2 cells.

To test the role of Pita and ZIPIC in the recruitment of CP190, we examined CP190 binding in S2 cells where ZIPIC or Pita had been depleted using RNAi (Supplemental Fig. S5A,B). Although either protein was strongly depleted (Supplemental Figs. S1A, S5C), residual binding of Pita and ZIPIC to their sites was still observed. However, the amount of CP190 was greatly reduced at two of three Pita sites and at three of four ZIPIC sites. When CP190 was depleted using RNAi, the binding of Pita also noticeably decreased at two sites, whereas binding of ZIPIC remained unchanged. This suggests that the CP190 protein is required for Pita binding to at least some of the tested sites, whereas in most cases, CP190 binding is dependent on either Pita or ZIPIC.

### CP190 sites marked by Pita and ZIPIC binding are enriched with BEAF-32 and dCTCF, but are depleted of Su(Hw) and occur next to actively transcribed genes

**Figure 5.**
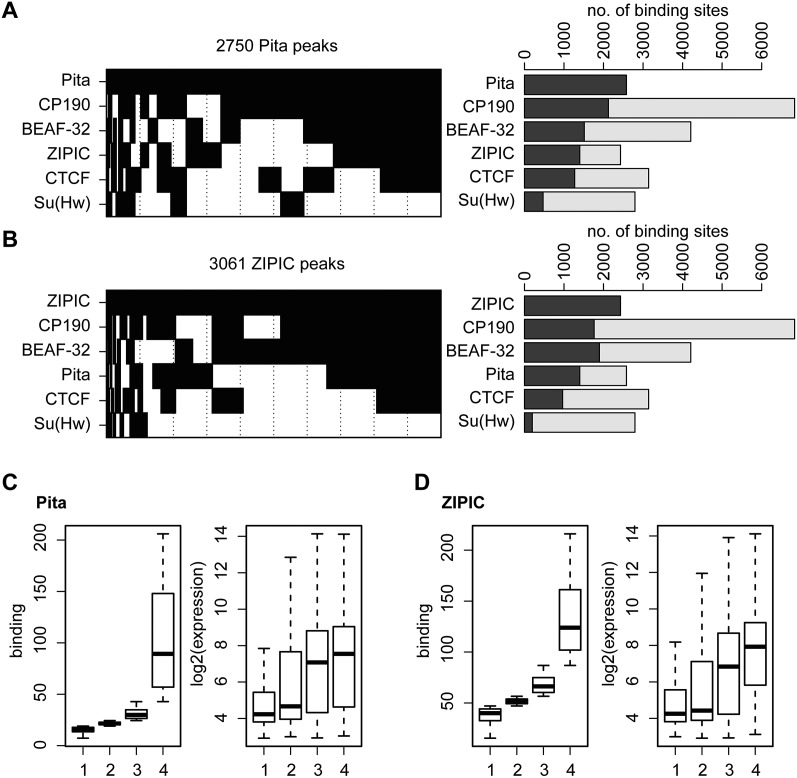
Pita and ZIPIC binding sites cluster with insulator factors. (*A*,*B*) Binary heat maps of (*A*) Pita and (*B*) ZIPIC binding sites classified into groups on the basis of their overlap with the binding of CP190, BEAF, dCTCF and Su(Hw). All sites bound by at least one of these factors are plotted (2579 sites for Pita and 2429 sites for ZIPIC). Each group is shown as a black rectangle in the corresponding row, and each column corresponds to a certain genomic location. The sites are sorted with respect to the occurrence frequency of a given binding pattern. The corresponding bar plot shows for each row the total number of peaks overlapping with the respective factor (dark gray). Additionally, the fraction of peaks not overlapping with Pita (*A*) or ZIPIC (*B*) is plotted in light gray. (*C*,*D*) For all RefGene transcription units, the maximum coverage for Pita (*C*) and ZIPIC (*D*) was calculated within a ±1-kb interval around the transcription start site, and the units were sorted accordingly into four groups, from (1) weak to (4) strong binding (*left* boxplot). The corresponding gene expression levels are shown in the *right* boxplot.

Several DNA-binding proteins, including BEAF-32, dCTCF, and Su(Hw), have recently been described as insulator factors that are capable of interacting with CP190. As shown in several studies, CP190 often occurs together with BEAF-32 and dCTCF or, alternatively, in combination with Su(Hw) and in some cases with Mod(mdg4) (Bushey et al. 2009; Nègre et al. 2010, 2011; Schwartz et al. 2012). When we examined all Pita and ZIPIC peaks overlapping with at least one other factor (Figs. 5A,B; Supplemental Fig. S6), it became evident that both Pita and ZIPIC tend to be in the group clustering with BEAF-32, CTCF, and CP190, whereas an overlap with Su(Hw) takes place only in a minority of cases. In fact, most of the Pita or of the ZIPIC binding sites are clustered with at least one other DNA binding factor, CTCF or BEAF-32. This supports the observation that cooperativity of these factors in tandemly aligned insulator factors is an important feature (Van Bortle et al. 2012, 2014).

Taking into account data on the association of BEAF-32 and CP190 with actively transcribed genes (Bartkuhn et al. 2009; Jiang et al. 2009; Yang et al. 2012), we were interested in finding out whether there is a correlation between transcription levels and Pita or ZIPIC binding. Therefore, we calculated the maximum coverage within an interval of –1 to +1 kb around the transcription start site for each transcription unit and sorted the units accordingly into four equal-sized groups. The results for both Pita and ZIPIC showed that the expression levels of the respective transcription units scaled up with an increase in binding (Fig. 5C,D).

To obtain further evidence for the relationship between CP190, Pita, and ZIPIC, we included the complete set of modENCODE ChIP-chip data in a comparative analysis (The modENCODE Consortium et al. 2010). We first compared the Pita and ZIPIC peaks to the binding regions recorded in all modENCODE experiments, calculated the ratio between the observed and expected numbers of overlapping peaks, and sorted the respective lists accordingly. Indeed, we found CP190 amongst the top ranked peak sets. Interestingly, other insulator-binding factors, such as dCTCF and BEAF-32, were also found in the highest-ranking peak sets (Supplemental Fig. S7). This finding strengthens the above result that Pita and ZIPIC are strongly linked with CP190 as well as other related insulator factors, such as BEAF and CTCF.

Similarly, we performed a more quantitative comparison between the binding profiles of Pita/ZIPIC and all profiles obtained by the modENCODE Consortium. We determined the average binding across the union of the two peak sets for each individual comparison under investigation and calculated the correlation coefficient across the unified peak set. Again, we ranked the factors accordingly and found that in the case of the Pita comparison, the highest degree of correlation was with chromatin insulators, with CP190 again appearing in the top ranked peak sets (Supplemental Fig. S8).

Taken together, these data show that Pita and ZIPIC have a much higher degree of overlap with CP190 than most other factors included in the modENCODE database.

### Testing Pita and ZIPIC binding sites in enhancer-blocking and anti-silencing assays in transgenic *Drosophila* lines

To test whether Pita and ZIPIC can function like known insulator proteins, we prepared DNA fragments containing five consensus binding sites for Pita (P^×5^) and four such sites for ZIPIC (Z^×4^). An at least threefold reiteration of the respective consensus sequence is found in ∼5%–10% of binding peaks (data not shown). The binding of Pita and ZIPIC to these sites was confirmed in vitro by EMSA (Supplemental Fig. S9A,B).

**Figure 6.**
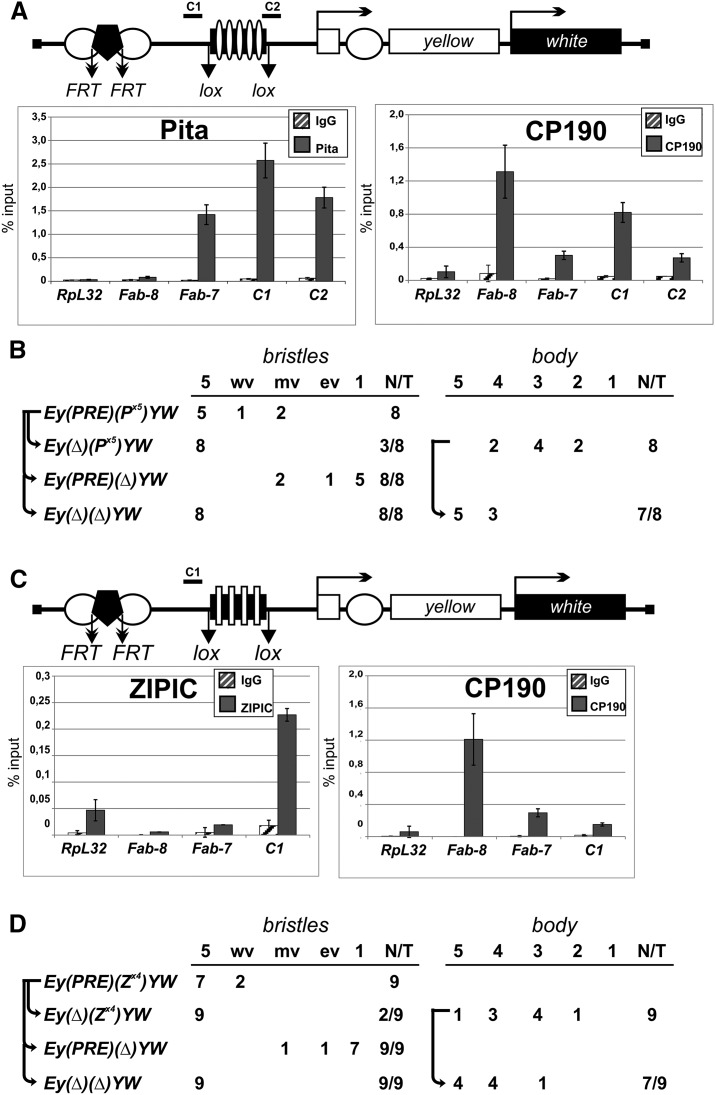
Testing Pita and ZIPIC binding sites for boundary and enhancer-blocking activities. (*A*,*C*) Reductive schemes of transgenic construct used to examine the boundary and enhancer-blocking activities of Pita (*A*) and ZIPIC (*C*) binding sites. The *yellow* and *white* genes are shown as boxes with arrows indicating the direction of their transcription. *Downward* arrows indicate target sites for Flp recombinase (FRT) or Cre recombinase (*lox*). The box with five white ovals (*A*) or four white rectangles (*C*) shows five Pita or four ZIPIC binding sites, respectively. White circles show the body and wing enhancers of the *yellow* gene; the black pentagon shows PRE from the *Ubx* gene. Histograms show the binding of (*A*) Pita and CP190 to Pita binding sites and (*C*) ZIPIC and CP190 to ZIPIC binding sites in the transgenic construct. Chromatin was isolated from transgenic flies carrying the construct and treated with antibodies to Pita, ZIPIC, and CP190. Nonspecific IgG was used as a negative control. The results of ChIP are presented as a percentage of input DNA. Relative locations of primers for ChIP are indicated at the construct scheme as C1 and C2. The *RpL32* coding region (devoid of binding sites for the test proteins) was used as a negative control; *Fab-8* and *Fab-7* as CP190-binding regions and *Fab-7* as a Pita-binding region were used as positive controls. Error bars indicate standard deviations of quadruplicate PCR measurements. (*B*,*D*) Experimental evidence that (*B*) Pita and (*D*) ZIPIC binding sites have an insulator activity. The “bristles” and “body” columns show the numbers of transgenic lines with different levels of pigmentation in corresponding cuticle structures. The “bristle” column shows degree of *yellow* expression in bristles of the thorax and head. The pigmentation was scored using a 5-point scale, where 1 denotes loss of pigmentation in all bristles on the thorax and head; (ev) extreme variegation (only 1–3 bristles on the thorax and head are partially pigmented); (mv) moderate variegation (about half of bristles are yellow); (wv) weak variegation (only 1–3 bristles on the thorax and head are yellow or partially pigmented); and (5) pigmentation of all bristles as in wild-type flies. The “body” column shows the numbers of transgenic lines with the *yellow* pigmentation level in the abdominal cuticle (reflecting the activity of the body enhancer); in most of the lines, the pigmentation level in wing blades (reflecting the activity of the wing enhancer) closely correlated with these scores. The level of pigmentation (i.e., of *y* expression) was estimated on an arbitrary five-grade scale, with wild-type expression and the absence of expression assigned scores 5 and 1, respectively (see Supplemental Fig. S10). In the N/T ratio, N is the number of transgenic lines in which flies acquired a new *yellow* phenotype after the deletion of a DNA fragment flanked by either FRT or *lox* sites, and T is the total number of examined transgenic lines.

To test the ability of Pita and ZIPIC binding sites to block enhancers and silencers, we used an assay in transgenic lines carrying the *yellow* reporter gene (Fig. 6A,C; Supplemental Fig. S9C), that is responsible for dark pigmentation of the larval and adult cuticle and its derivatives (Supplemental Fig. S10). Two upstream enhancers stimulate its expression in the body cuticle and wing blades, whereas the enhancer responsible for *yellow* activation in bristles is located in the intron (Geyer and Corces 1987). As a silencer, we chose the 661-bp Polycomb response element (PRE) from the regulatory region of homeotic gene *Ultrabithorax* (*Ubx*), which is often used in anti-silencing assays (Sigrist and Pirrotta 1997; Mallin et al. 1998; Comet et al. 2006). The PRE flanked by FRT sites was inserted between the wing and body enhancers at −1870 relative to the *yellow* transcription start site. The tested DNA fragments flanked by *lox* sites were inserted at −893 between the *yellow* promoter and the regulatory region including PRE and the enhancers (Fig. 6A,C). Hereinafter, parentheses in construct designations enclose the elements flanked by the FRT or *lox* sites at which these elements can be excised by crossing with flies expressing Flp or Cre recombinase (as described in Supplemental Methods).

ChIP with chromatin isolated from pupae confirmed that Pita and CP190 bind to the P^×5^ fragment (Fig. 6A), and that ZIPIC and CP190 bind to the Z^×4^ fragment (Fig. 6C). In all transgenic lines, Pita and ZIPIC binding sites protected *yellow* expression in bristles from PRE-mediated repression. Moreover, these sites also partially blocked the *yellow* enhancers in the absence of PRE (Fig. 6B,D). Similar results had been previously obtained using binding sites for the known insulator proteins Su(Hw) and Dwg (also known as Zw5) (Gaszner et al. 1999; Scott et al. 1999; Golovnin et al. 2003; Kyrchanova et al. 2008a, 2013). Thus, Pita and ZIPIC function like insulator proteins by blocking enhancers and protecting the reporter gene expression from PRE-mediated silencing.

In Schwartz et al. (2012) it was reported that the 1-kb region (100C) bound by CP190 effectively blocks communication between the *yellow* enhancers and the promoter. This region contains a binding site for Pita (Supplemental Figs. S4, S5A), but not for other known insulator proteins. To test whether Pita is essential for enhancer blocking activity of the 100C region, we tested the derivative 372-bp fragment (d100C) that includes a Pita site in the enhancer blocking assay. The d100C fragment flanked by *lox* sites was inserted between the *yellow* enhancers and the promoter (Supplemental Fig. S11A). The 372-bp region effectively blocked the *yellow* enhancers in nine independently obtained transgenic lines. The binding of Pita and CP190 to the d100C in the transgenic construct was confirmed by immunoprecipitation of chromatin isolated from pupae (Supplemental Fig. S11B). To confirm the role of Pita in enhancer blocking activity, we mutated its binding site in the d100C fragment (d100C^m^). The mutated fragment failed to block the *yellow* enhancers in four independently obtained transgenic lines (Supplemental Fig. S11C). ChIP showed that neither Pita nor CP190 bound to d100C^m^ in transgenic pupae. Thus, the Pita/CP190 complex is critical for enhancer blocking activity of the 100C region.

### Pita in cooperation with dCTCF contributes to the activity of *MCP* insulator

Our results indicate that Pita and ZIPIC frequently colocalize with dCTCF. In particular, Pita binds in close proximity to the dCTCF binding site in the *MCP* insulator (Supplemental Fig. S12A). In *Drosophila*, dCTCF and Pita are recruited to the *MCP* region in flies (Supplemental Fig. S12B) and bind to the DNA fragment corresponding to the *MCP* insulator in vitro (Supplemental Fig. S12C). To test whether Pita contributes to the activity of the *MCP* insulator, we used the above assay in transgenic lines.

**Figure 7.**
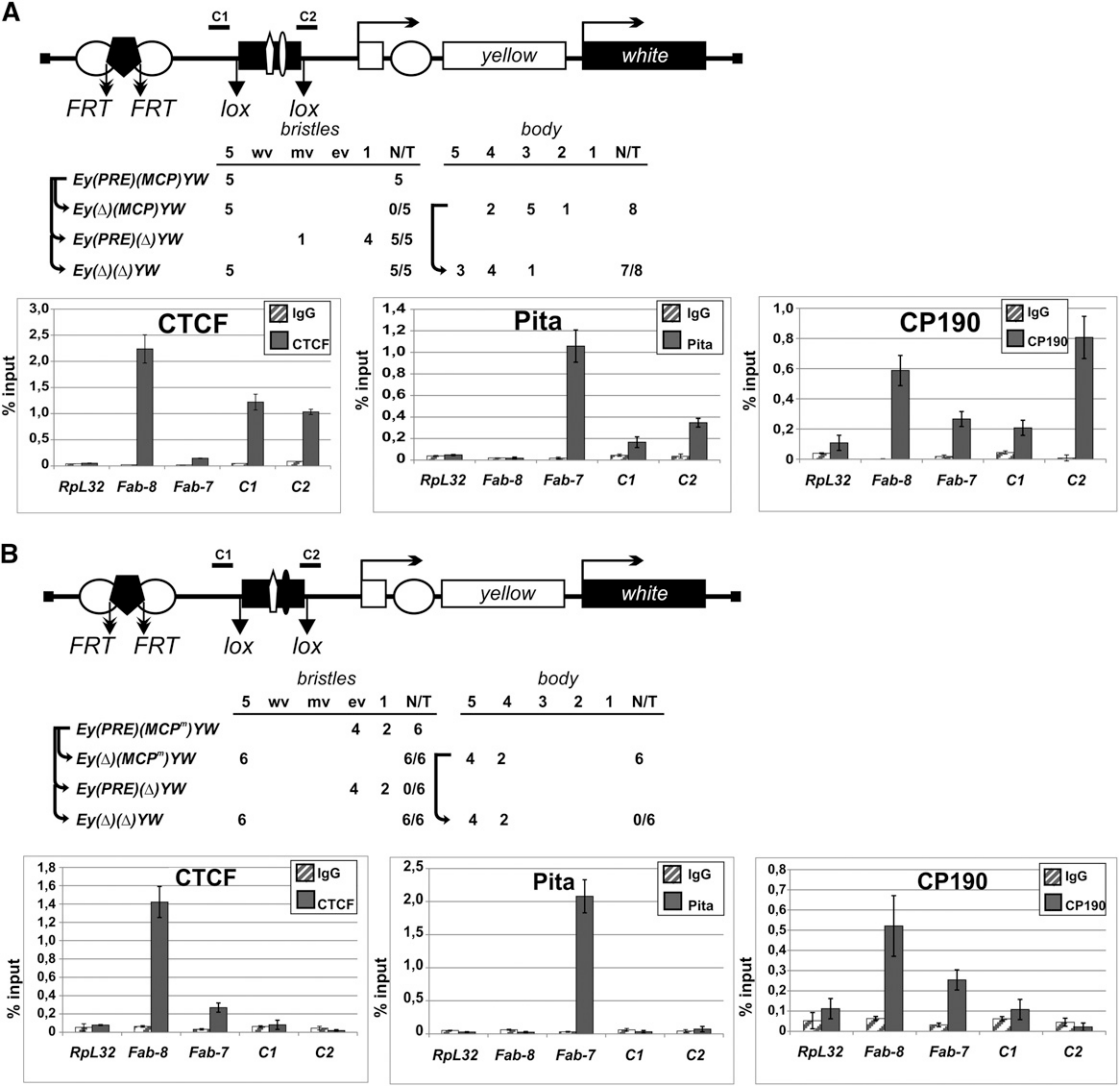
Testing the enhancer-blocking activity of Pita binding sites at the *MCP* insulator. Reductive schemes of transgenic constructs used to examine the enhancer-blocking activity of Pita binding sites at the *MCP* element. This element is shown as a black box with a white pentagon (dCTCF binding site) and a white or a black oval indicating wild-type (*A*) or mutated (*B*) Pita binding sites. Figures in the columns show the numbers of transgenic lines with different levels of pigmentation in the abdominal structures. Histograms show binding of dCTCF, Pita, and CP190 proteins to *MCP* with wild-type (*A*) or mutated (*B*) Pita binding sites in the transgenic construct. For other designations, see Figure 6.

As we had found previously, the *MCP* insulator partially blocks the yellow enhancer (Gruzdeva et al. 2005). To further test the activity of *MCP*, we made transgenic lines in which the 340-bp *MCP* insulator flanked by *lox* sites was inserted between the regulatory elements (the enhancers and PRE) and the *yellow* promoter. We found that the *MCP* insulator protected *yellow* expression in bristles from repression in all five transgenic lines with active PRE silencer and, in addition, partially blocked the *yellow* enhancers in the absence of PRE (Fig. 7A). The binding of dCTCF, Pita, and CP190 to the 340-bp *MCP* in the transgenic construct was confirmed by immunoprecipitation with chromatin isolated from pupae (Fig. 7A).

Next, we mutated the binding site for Pita in the *MCP* insulator (*MCP*^m^). The results of electrophoretic mobility shift assay confirmed that dCTCF bound to *MCP*^m^, whereas Pita did not (Supplemental Fig. S12C). In transgenic lines, *MCP*^m^ failed to protect *yellow* expression from PRE-mediated silencing or to block the *yellow* enhancers (Fig. 7B). ChIP showed that neither dCTCF nor Pita bound to *MCP*^m^ in transgenic pupae (Fig. 7B). These results provide strong evidence that Pita and dCTCF cooperate in binding to the *MCP* insulator and contribute to its activity.

## Discussion

CP190 is known to interact with promoters and with the insulator proteins dCTCF, BEAF-32, and Su(Hw). Additional IBPs have been postulated, based on previous mapping results of the CP190 factor binding to additional sites (Schwartz et al. 2012). Here we describe two new proteins, Pita and ZIPIC, that interact with CP190 and display insulator function. Within Drosophilidae, these zinc-finger proteins are highly conserved, but not outside of Diptera. CP190 is recruited to model binding sites for Pita and for ZIPIC as well as to endogenous sites, indicating that both participate in targeting of CP190 to chromatin. Indeed, our studies on three endogenous Pita and four ZIPIC binding sites show that CP190 binding depends on the presence of Pita or ZIPIC.

Pita or ZIPIC interact directly with CP190, as demonstrated by mass spectrometric analysis of CP190 complexes, by coimmunoprecipitation, and by yeast two-hybrid analysis. Protein interaction requires the BTB domain of CP190 to bind the Pita domain located between the ZAD and zinc-finger domains. Thus, the BTB domain of CP190 is required for interactions with insulator proteins, which is in agreement with the previous observation that only the BTB domain with the adjacent aspartic acid-rich D-domain are required for the association of CP190 with polytene chromosomes (Oliver et al. 2010). The BTB domain forms stable dimers (Bonchuk et al. 2011) and is similar in structure to the BTB domain of human ZBTB33 (also known as Kaiso), which interacts with CTCF (Defossez et al. 2005). Similar to CP190, ZBTB33 associates with chromatin during interphase and with centrosomes in mitotic cells (Soubry et al. 2010). It is noteworthy that the centrosomal targeting domain in both ZBTB33 and CP190 is adjacent to zinc fingers. Thus, CP190 and ZBTB33 appear to have partially overlapping functions in the regulation of transcription and in the activity of insulators. In contrast to Pita, the ZIPIC protein interacts with the centrosomal targeting domain (M domain) of CP190. Therefore, two different domains of CP190 are involved in interactions with DNA-binding proteins.

For several insulator factors, a substantial fraction of the protein is associated with active promoters. This has been shown for CP190 (Bartkuhn et al. 2009), for dCTCF (Nègre et al. 2010), and for BEAF-32 (Bushey et al. 2009; Jiang et al. 2009; Nègre et al. 2010; Yang et al. 2012). Similarly, we find a strong correlation between Pita and ZIPIC binding to transcriptional start sites and gene activity. Indeed, it has been postulated that promoters and insulators are functionally and evolutionary related (Geyer 1997; Raab and Kamakaka 2010).

In accordance with the role of insulator proteins in the formation of boundaries between active and repressed chromatins, dCTCF and CP190 are associated with PcG domains throughout the genome (Bartkuhn et al. 2009; Nègre et al. 2010, 2011). It has been shown that the H3K27me3 domain boundaries correspond to dCTCF sites that are cobound by CP190 (Bartkuhn et al. 2009; Schwartz et al. 2012). However, inactivation of dCTCF has only a limited effect on the spreading of H3K27me3. To explain these ambiguous results, it has been suggested that insulator proteins have multiple functions at chromatin boundaries and that additional insulator factors may bind as well. Our results show that Pita and ZIPIC binding sites can block the spreading of PRE-mediated silencing. Thus, it appears that after dCTCF knockdown, other insulator proteins that are bound next to dCTCF can fulfil a barrier function at numerous boundaries of chromatin domains.

This argues for a frequent clustering of insulator proteins, which indeed has been shown for CP190, dCTCF, and BEAF-32, whereas Su(Hw) is in most cases not involved in these clusters (Schwartz et al. 2012; Van Bortle et al. 2012, 2014). Similarly, for Pita and ZIPIC, we find a high percentage (95% of Pita sites and 77% of ZIPIC sites) to be clustered with at least one other IBP. These clusters, with each of the DNA-bound factors contacting CP190 molecules, which by themselves can dimerize, may explain the strong correlation of binding affinity we have observed. High affinity binding sites for Pita and ZIPIC are simultaneously high affinity sites for CP190. This may lead to an interdependency seen when CP190 is depleted. Depending on the insulator studied, three different situations can be envisaged: (1) Depletion of a single DNA bound factor has no consequences on CP190 binding, as the remaining insulator proteins are sufficient for CP190 binding. This has been observed for BEAF-32 depletion, which does not affect CP190 recruitment to chromatin (Schwartz et al. 2012; Lim et al. 2013). (2) At other sites, CP190 may require the cooperation with at least two DNA-bound IBPs. This idea is supported in the case of Pita and ZIPIC, since different CP190 domains are contacted by each protein. Functionally, we have observed this situation with the *MCP* insulator. Pita and dCTCF proteins bind to adjacent sites in the *MCP* insulator (Gruzdeva et al. 2005; Kyrchanova et al. 2007). Two *MCP* insulators interact in an orientation-dependent manner and can support super-long-distance interactions between transgenes (Muller et al. 1999; Vazquez et al. 2006; Kyrchanova et al. 2007, 2011). These properties of the *MCP* insulator are explained by the binding of several insulator proteins that support specific long-distance interactions (Kyrchanova and Georgiev 2014). When we destroy the binding site for Pita, clearly the protection from PRE mediated repression is impaired, suggesting that the cooperation between Pita and dCTCF in CP190 binding is lost. (3) Cooperation at clustered binding sites may help IBPs with low DNA-binding affinity to bind efficiently within the cluster. Upon CP190 depletion, cooperation is lost, and the weak DNA-binding may cause the loss of particular low-affinity DNA-binding factors. Such an interdependency has been found for CP190 and Su(Hw) (Schwartz et al. 2012). Similarly, we have observed such an effect with two of three tested Pita binding sites losing Pita binding upon CP190 depletion.

Therefore, the roles of individual proteins in the formation of insulator complexes on chromatin are primarily dependent on a given combination of binding sites for insulator proteins as well as on the contacted sites in long-range interactions.

In conclusion, our results show that the number of insulator-like proteins in the *Drosophila* genome is greater than previously thought. These proteins interact with CP190 and may have multiple functions in organizing chromosome architecture.

## Methods

### Protein expression and purification

Recombinant proteins were expressed in *E. coli* BL21 cells and purified using standard procedures. Briefly, the cells expressing Pita[99–302aa], Pita[550–683aa], ZIPIC[84–257aa] were disrupted by sonication in buffer A (40 mM HEPES-KOH, pH 7.7; 400 mM NaCl, 5 mM β-mercaptoethanol, 0.1% NP-40, 20 mM imidazole, 1 mM PMSF, 1:1000 Complete Protease Inhibitor Cocktail VII [Calbiochem]). The lysate was cleared by centrifugation and applied onto a Ni-NTA (Pierce) column. After washing, the bound proteins were eluted with 300 mM imidazole and dialyzed against appropriate buffer.

Full-length dCTCF, Pita, and ZIPIC and their zinc-finger domains were expressed as fusions with MBP. The cells expressing these proteins were disrupted as described above in buffer A with the addition of 0.1 mM ZnCl_2_. The lysate was applied onto an immobilized amylose (New England Biolabs) column in starting buffer (20 mM Tris-HCl, pH 8.0; 20 mМ KCl, 100 mМ NaCl, 5 mМ MgCl_2_, 100 mМ ZnCl_2_, 10% glycerol, 0.1% NP-40, 0.5 mМ PMSF, 1% β-mercaptoethanol, 1:1000 Calbiochem Cocktail VII). After washing, the bound proteins were eluted with maltose-containing buffer (20 mМ Tris-HCl, pH 7.4; 200 mМ NaCl, 0.1 mМ ZnCl_2_, 10 mМ maltose, 1% β-mercaptoethanol) and dialyzed against an appropriate buffer.

### Pull-down assays

For pull-down assays, we performed coexpression of full-length Pita and ZIPIC proteins fused with MBP or their zinc-finger domains fused with GST and of full-length CP190 and its [1–126] and [245–599] regions fused with 6 × His in *E. coli* BL21 cells. The cells were grown in LB medium at 37°C to an A_600_ of 1.0 and then induced with 1 mM IPTG overnight at 18°C. ZnCl_2_ was added to a final concentration of 200 μM prior to induction. The cells were then disrupted by sonication in buffer A (20 mM HEPES-KOH, pH 7.7; 150 mM NaCl, 5mM MgCl_2_, 0.1 mM ZnCl_2_, 0.1% NP-40, 10% (w/w) glycerol, 0.5 mM PMSF, 1 mM DTT, 1:1000 Calbiochem Cocktail VII) and centrifuged at 5000*g*. The supernatant was mixed with pre-equilibrated Ni-NTA resin (Pierce) (6 × His pull-down assay), glutathione resin (Pierce) (GST pull-down assay), or amylose resin (New England Biolabs) (MBP pull-down assay) and incubated for 20 min at room temperature with rotation. After binding, the resin was washed with four portions of buffer B (buffer A with 500 mM NaCl) and treated with elution buffer (GST-pull-down: 50 mM Tris, pH 8.0, with 200 mM NaCl and 30 mM glutathione; MBP-pull-down: 20 mM HEPES-KOH, pH 7.6, with 200 mM NaCl and 10 mM maltose; 6 × His-pull-down: 40 mM HEPES-KOH, pH 7.7, with 400 mM NaCl, 5 mM β-mercaptoethanol, and 300 mM imidazole) for 20 min. The mixture was then centrifuged at 2000 rpm for 1 min, and the supernatant was analyzed by SDS-PAGE with Coomassie staining.

### Preparation of embryonic nuclear extract

Nuclear extracts were prepared from 0- to 12-h embryos and used to immunoprecipitate the protein complexes of interest. For this purpose, 60 g of embryos 0–12 h were collected and processed as described (Kamakaka et al. 1991), with the following modifications. The nuclei were resuspended in Buffer I, layered upon the same volume of Buffer S (15 mM HEPES-KOH, pH 7.6; 10 mM KCl, 5 mM MgCl_2_, 0.1 mM EDTA, 0.5 mM EGTA, 0.8 M sucrose, 1 mM DTT, 0.5 mM PMSF, Calbiochem Cocktail V), and pelleted in a bucket rotor at 2500*g* for 20 min. At the final stage, the nuclear extract was precipitated with ammonium sulfate (0.3 g/mL extract), and the precipitate was dialyzed against HEMG-40 buffer (25 mM HEPES-KOH, pH 7.6; 40 mM KCl, 12.5 mM MgCl_2_, 0.1 mM EDTA, 10% glycerol, 1 mM DTT, 0.5 mM PMSF, Calbiochem Cocktail V), and frozen in liquid nitrogen. The lysate was used in immunoprecipitation assays (as described in Supplemental Methods).

### Yeast two-hybrid assay

Yeast two-hybrid assay was carried out using yeast strain pJ69-4A with plasmids and protocols from Clontech. For growth assays, plasmids were transformed into yeast strain pJ69-4A by the lithium acetate method, as described by the manufacturer, and plated on media without tryptophan and leucine. After 2 d of growth at 30°C, the cells were plated on selective media without tryptophan, leucine, histidine, and adenine, and their growth was compared after 2–3 d. Each assay was repeated three times.

Technical details for S2 cell nuclear lysate preparation and mass spectrometric analysis, RNA interference in *Drosophila* S2 cells, RNA isolation and real-time PCR, plasmid construction, antibodies used, immunostaining of polytene chromosomes, immunoprecipitation of protein nuclear extract, chromatin immunoprecipitation from S2 cells, chromatin immunoprecipitation from pupae and embryos, deep sequencing of ChIP DNA, bioinformatics analyses, electrophoretic mobility shift assay (EMSA), generation and analysis of transgenic lines are presented in Supplemental Methods*.*

## Data access

All data sets reported in this study have been submitted to the NCBI Gene Expression Omnibus (GEO; http://www.ncbi.nlm.nih.gov/geo/) under accession number GSE54337.

## Supplementary Material

Supplemental Material
