# Supplementary material for: Two new insulator proteins, Pita and ZIPIC, target CP190 to chromatin
Source: Genome Res. 2015 Jan;25(1):89–99. doi: 10.1101/gr.174169.114 (PMC4317163; doi:10.1101/gr.174169.114)
Supplement: Supplemental Material [file supp_25_1_89__index.html]

Two new insulator proteins, Pita and ZIPIC, target CP190 to chromatin — Two new insulator proteins, Pita and ZIPIC, target CP190 to chromatin — Supplemental Material 

# Two new insulator proteins, Pita and ZIPIC, target CP190 to chromatin

## Supplemental Material

**Files in this Data Supplement:**

- Supp FigS1.tif
- Supp FigS2\_1.tif
- Supp FigS2.tif
- Supp FigS3.tif
- Supp FigS4.tif
- Supp FigS6.tif
- Supp FigS7.tif
- Supp FigS8.tif
- Supp FigS9.tif
- Supp FigS10.tif
- Supp FigS11.tif
- Supp FigS12.tif
- Supplemental Material.docx
