# Supplementary figures and images for: Two new insulator proteins, Pita and ZIPIC, target CP190 to chromatin
Source: Genome Res. 2015 Jan;25(1):89–99. doi: 10.1101/gr.174169.114 (PMC4317163; doi:10.1101/gr.174169.114)

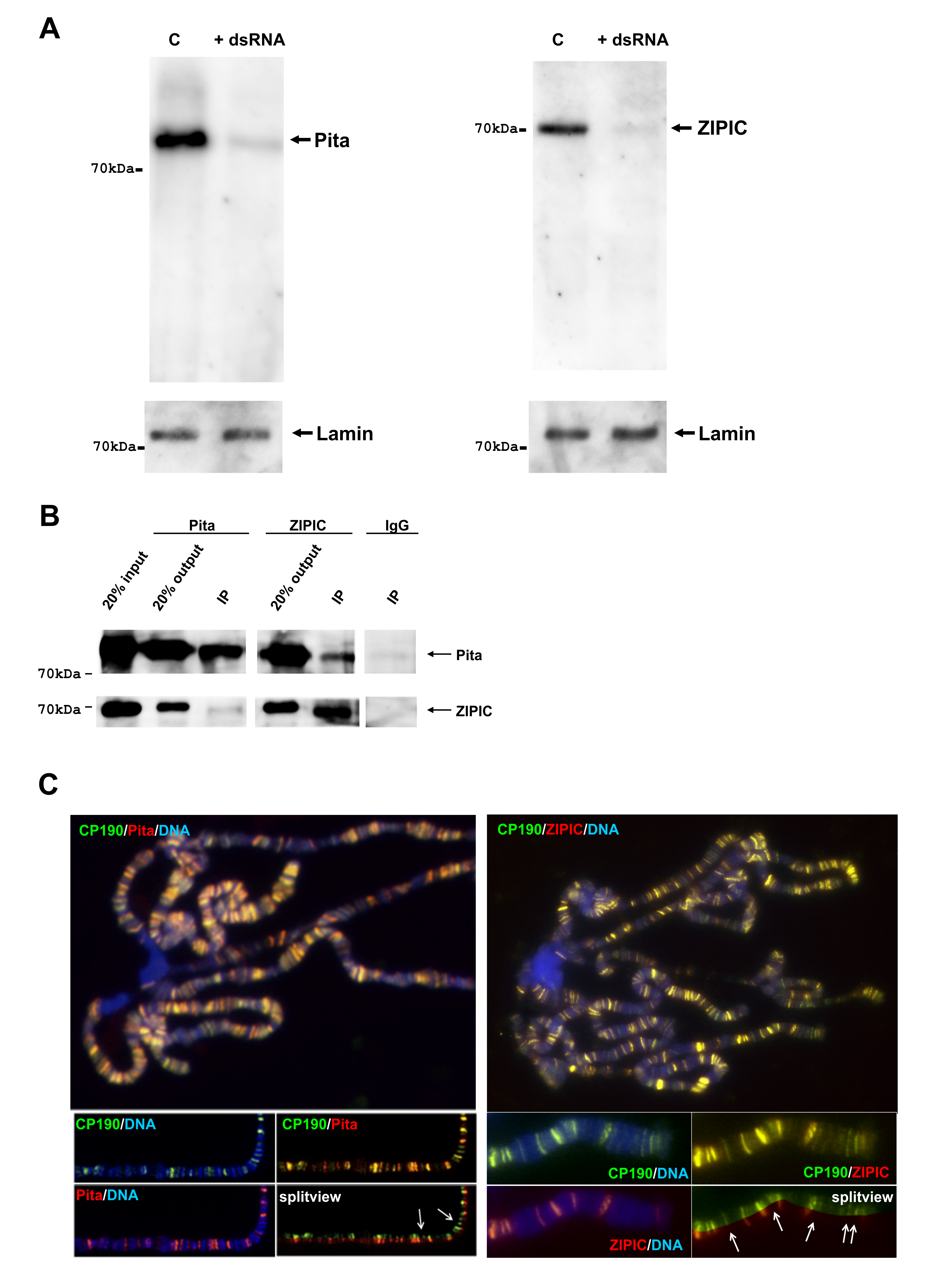

Supplement: Supplemental Material [file supp_gr.174169.114_Fig_S1.tif]

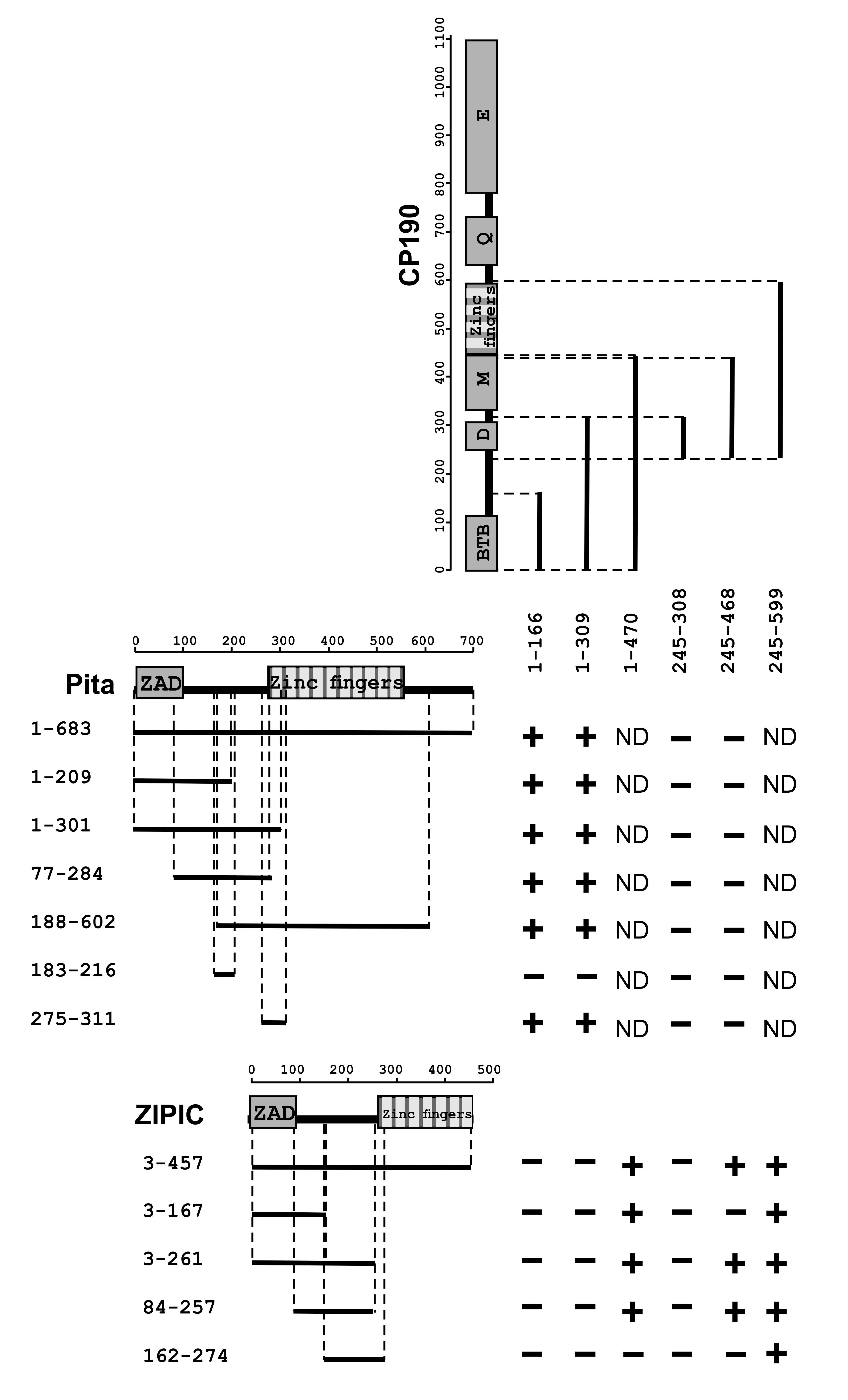

Supplement: Supplemental Material [file supp_gr.174169.114_Fig_S2_1.tif]

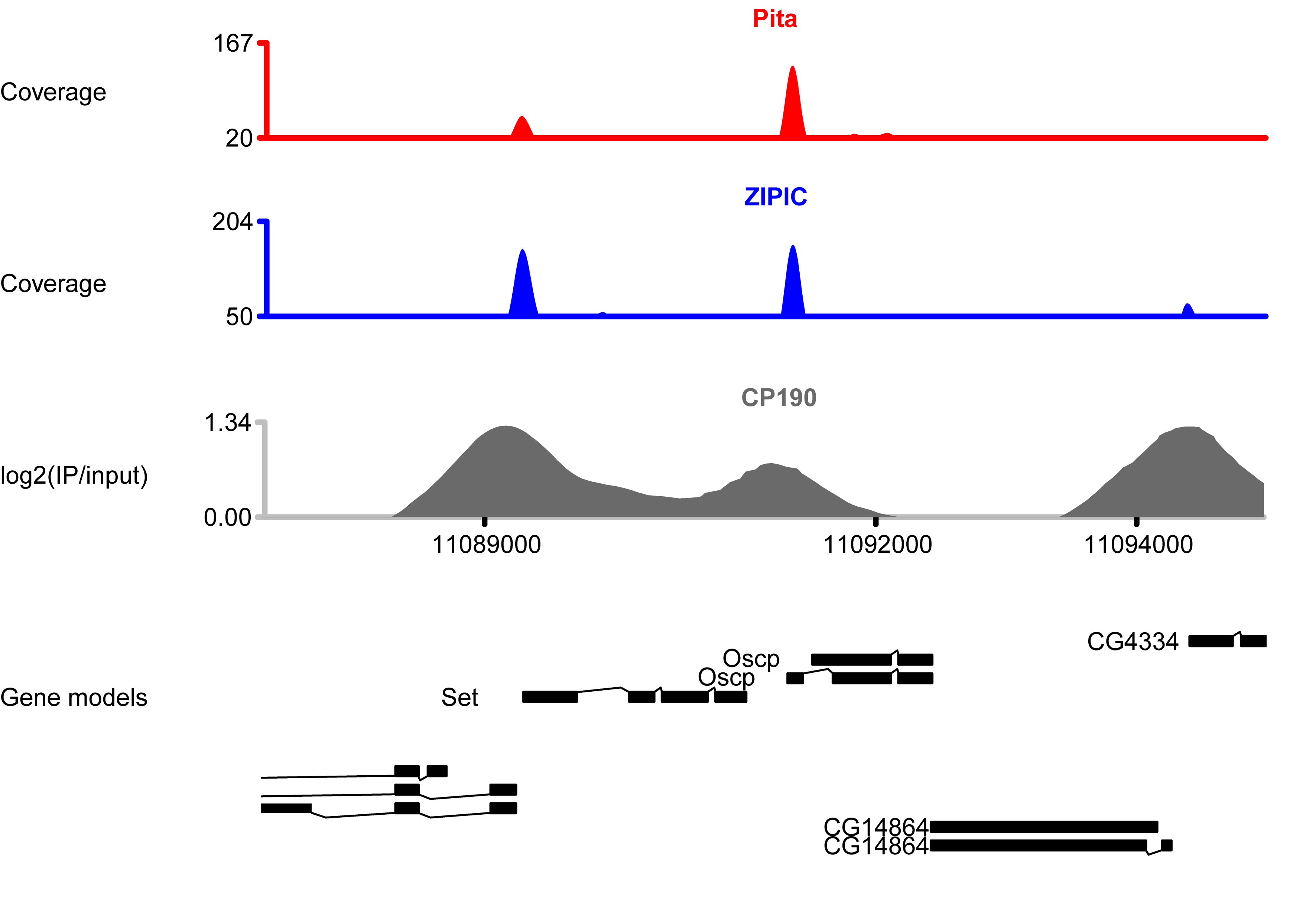

Supplement: Supplemental Material [file supp_gr.174169.114_Fig_S3.tif]

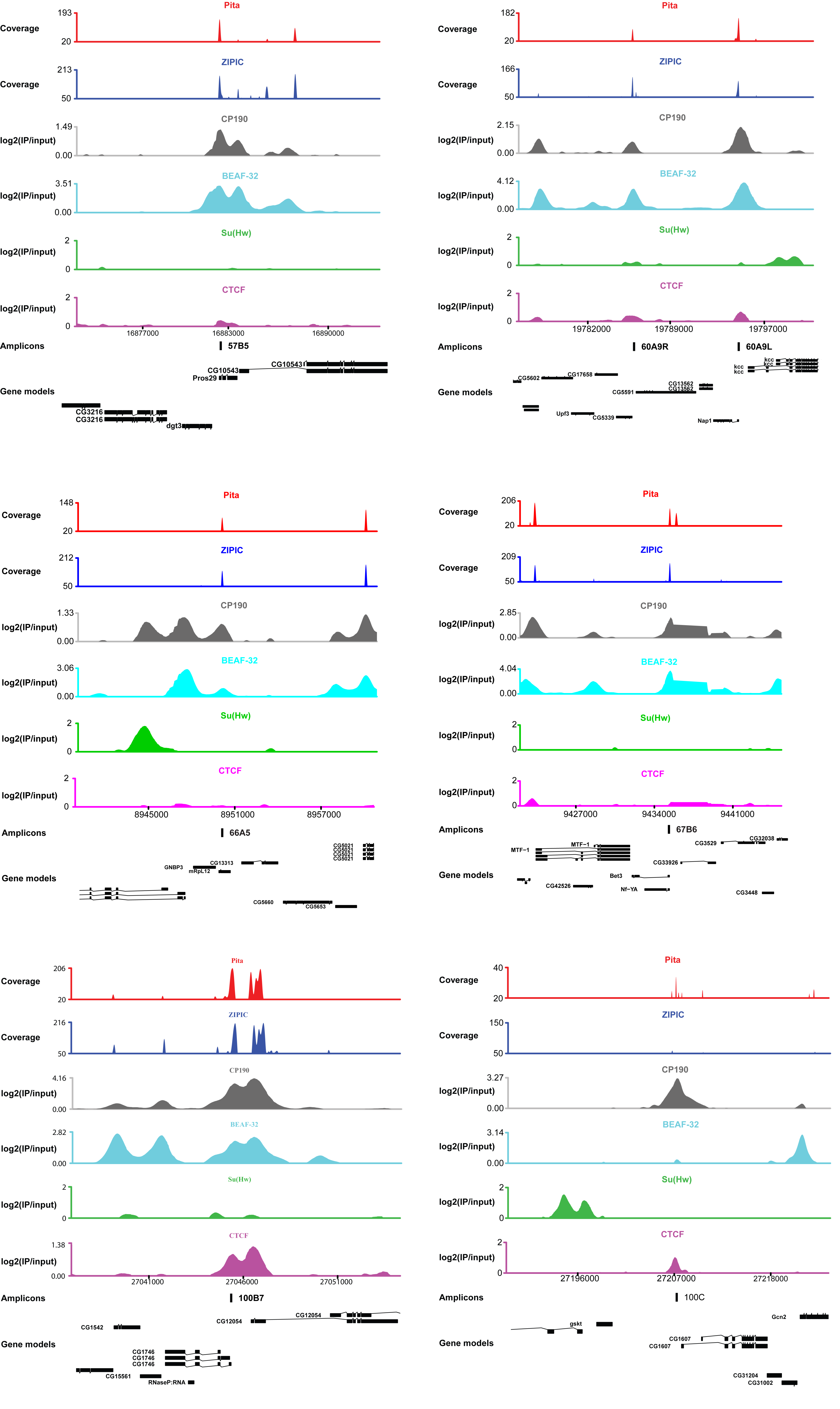

Supplement: Supplemental Material [file supp_gr.174169.114_Fig_S4.tif]

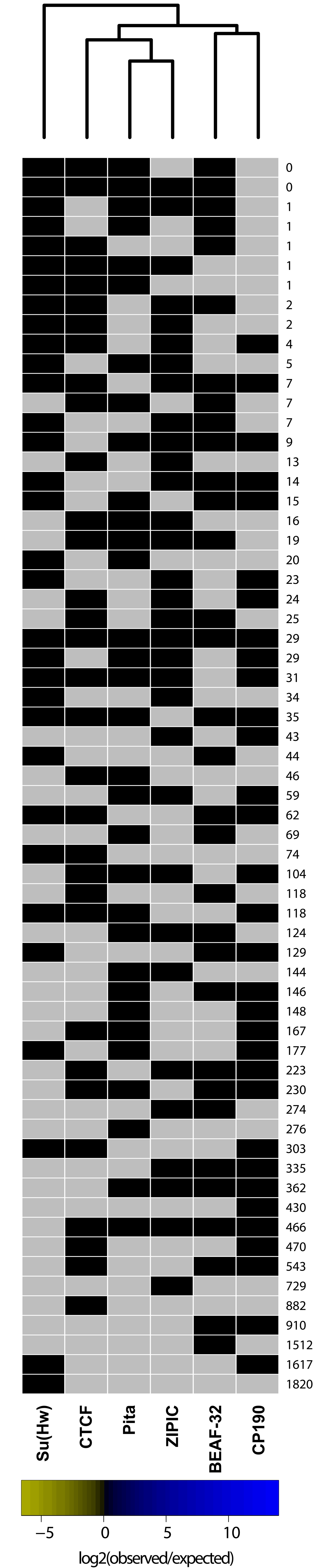

Supplement: Supplemental Material [file supp_gr.174169.114_Fig_S6.tif]

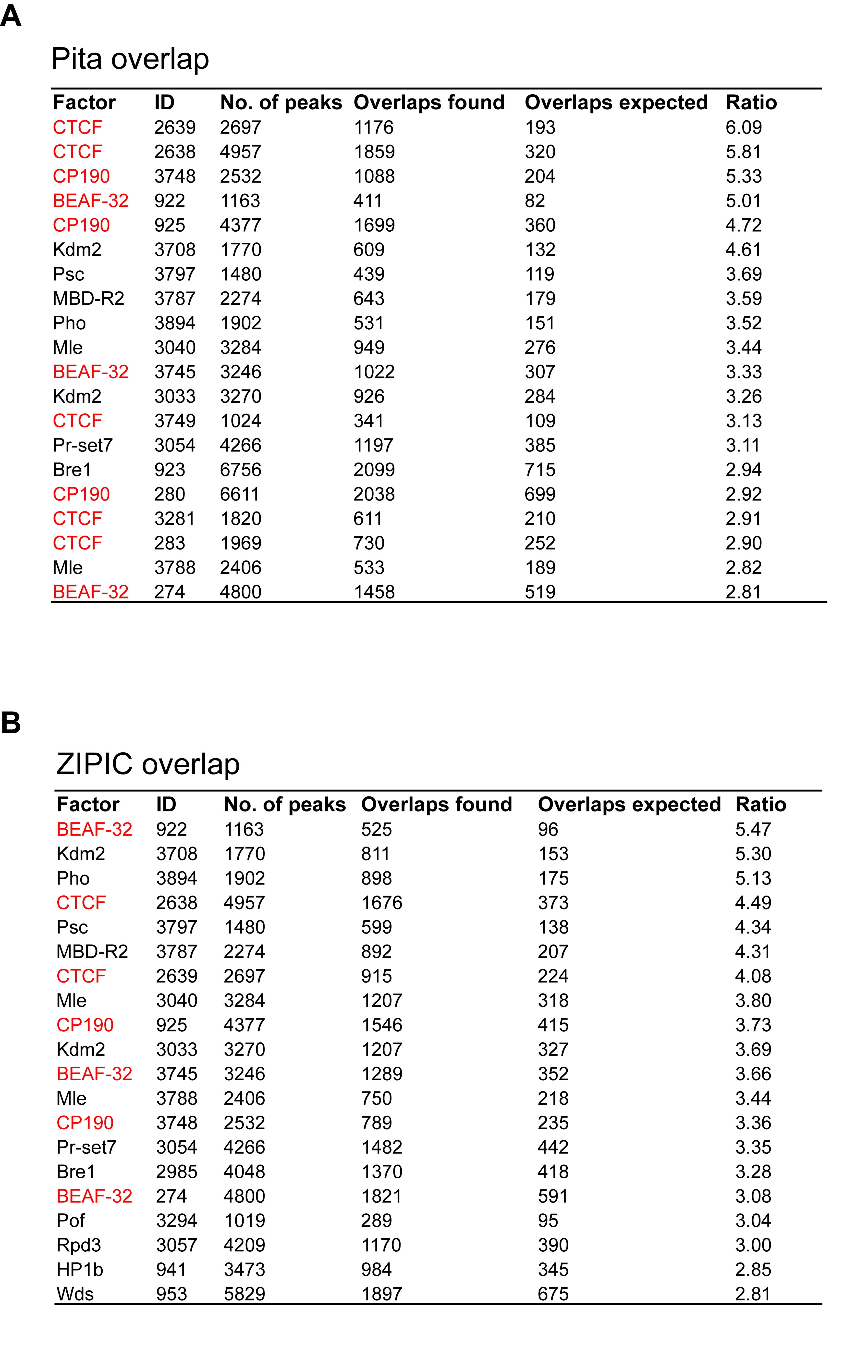

Supplement: Supplemental Material [file supp_gr.174169.114_Fig_S7.tif]

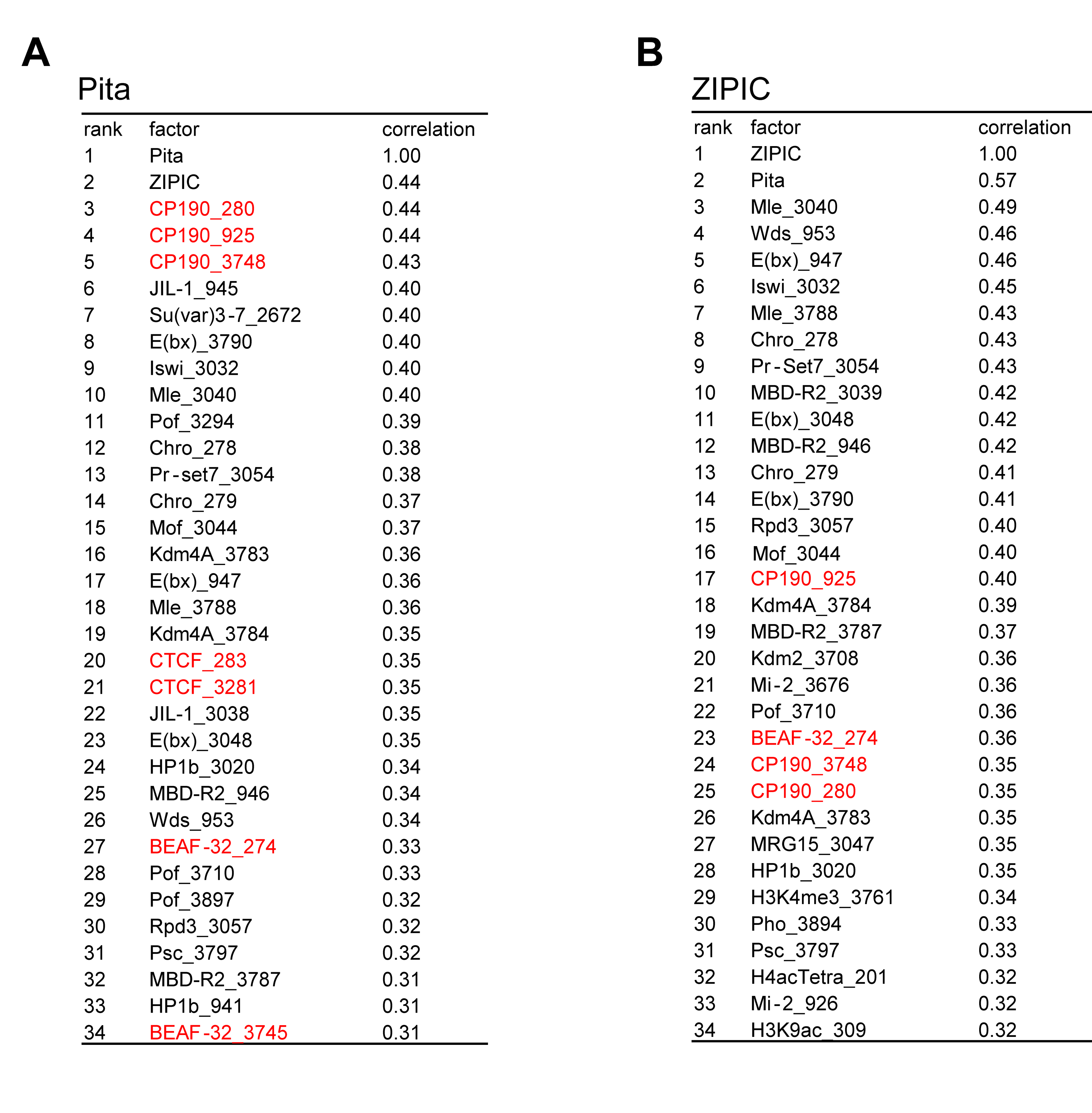

Supplement: Supplemental Material [file supp_gr.174169.114_Fig_S8.tif]

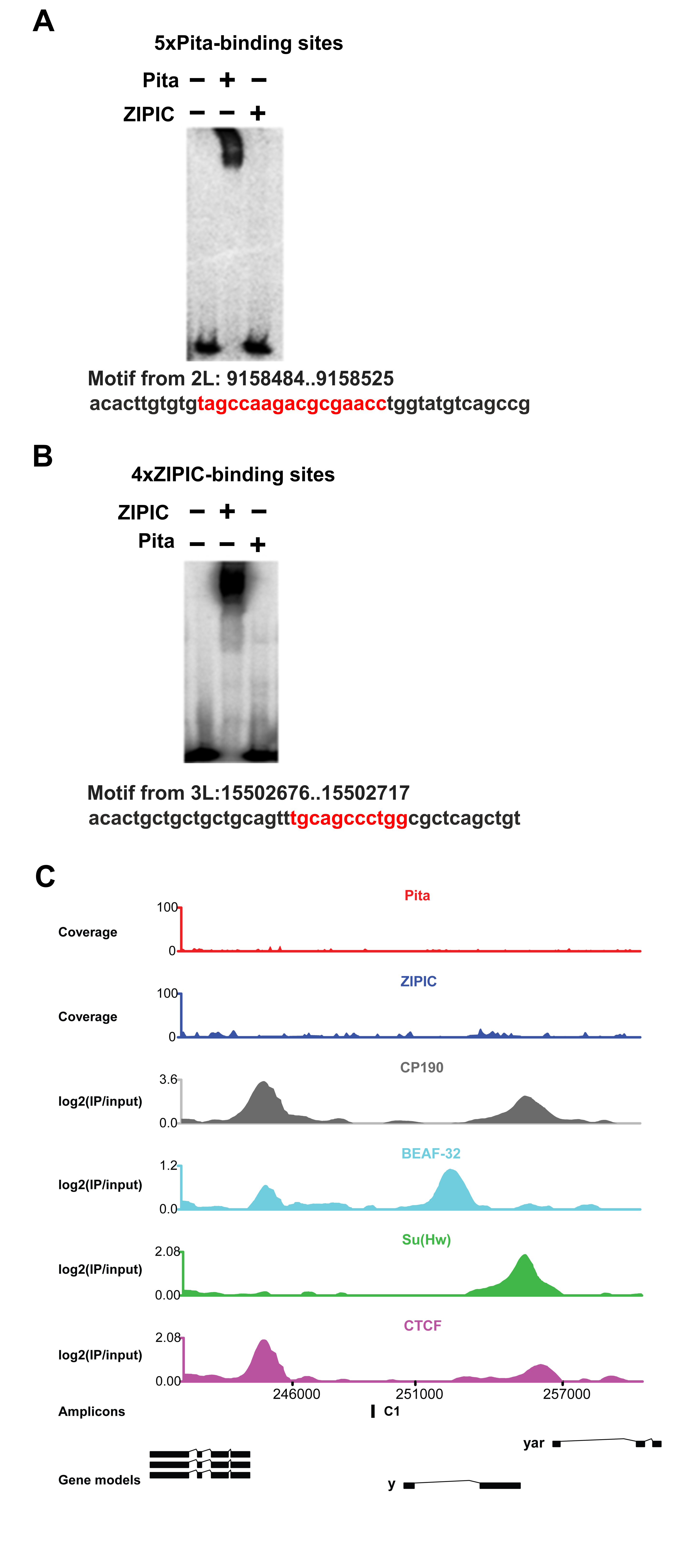

Supplement: Supplemental Material [file supp_gr.174169.114_Fig_S9.tif]

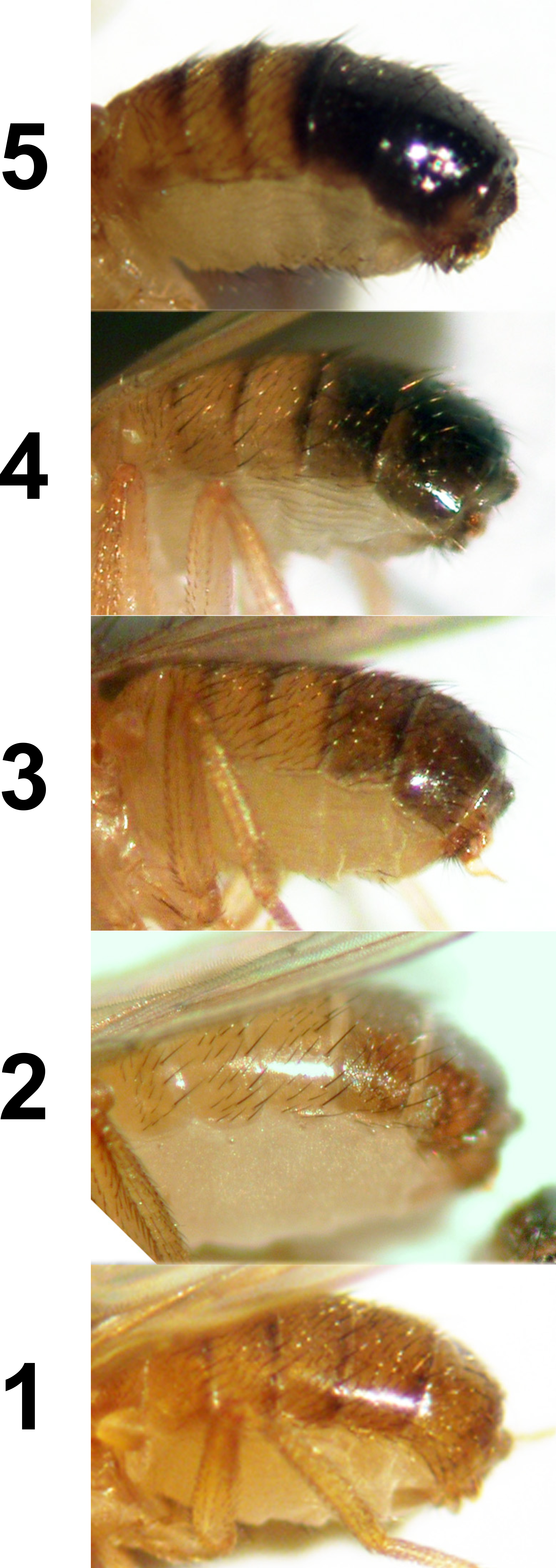

Supplement: Supplemental Material [file supp_gr.174169.114_Fig_S10.tif]

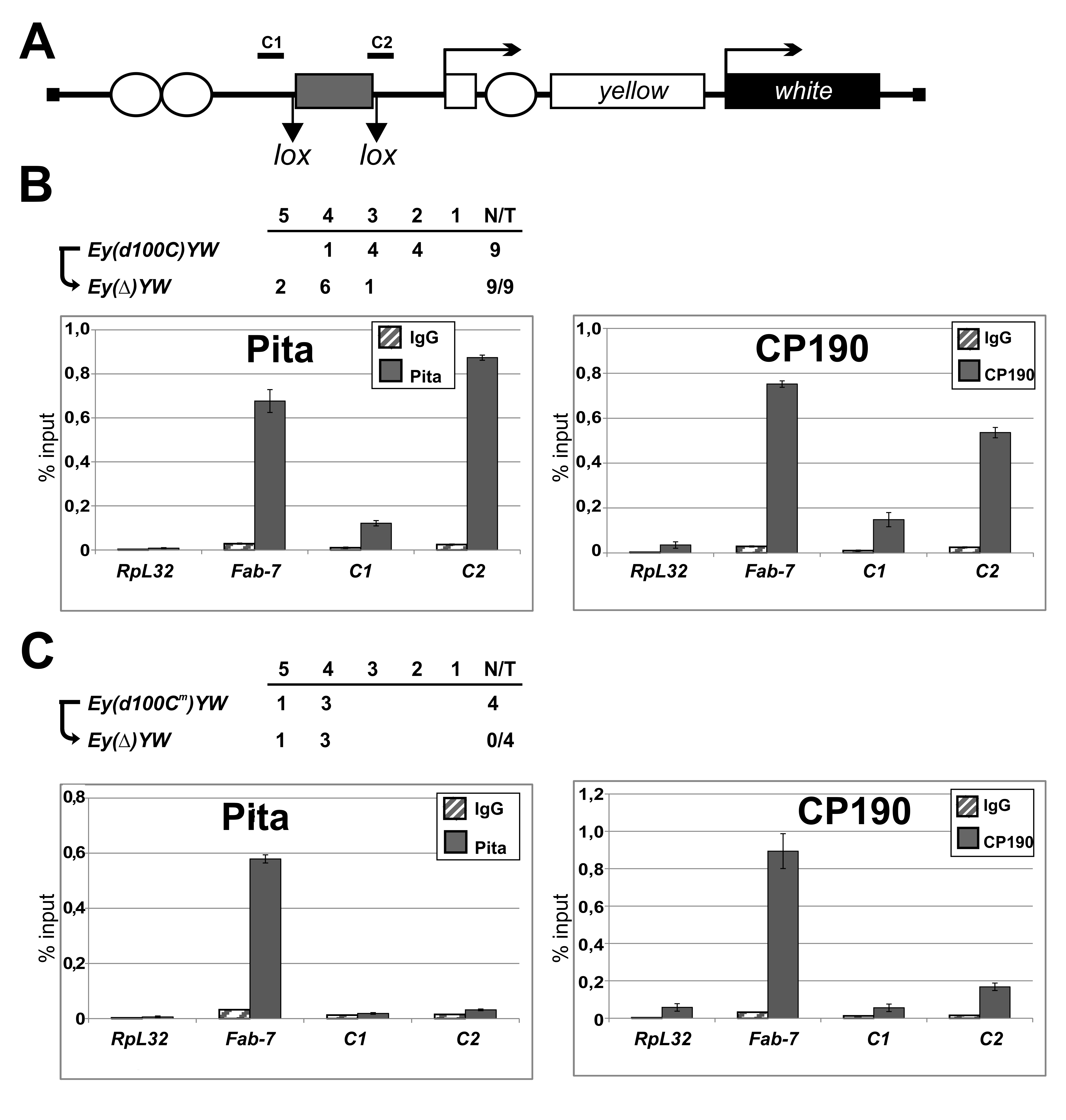

Supplement: Supplemental Material [file supp_gr.174169.114_Fig_S11.tif]

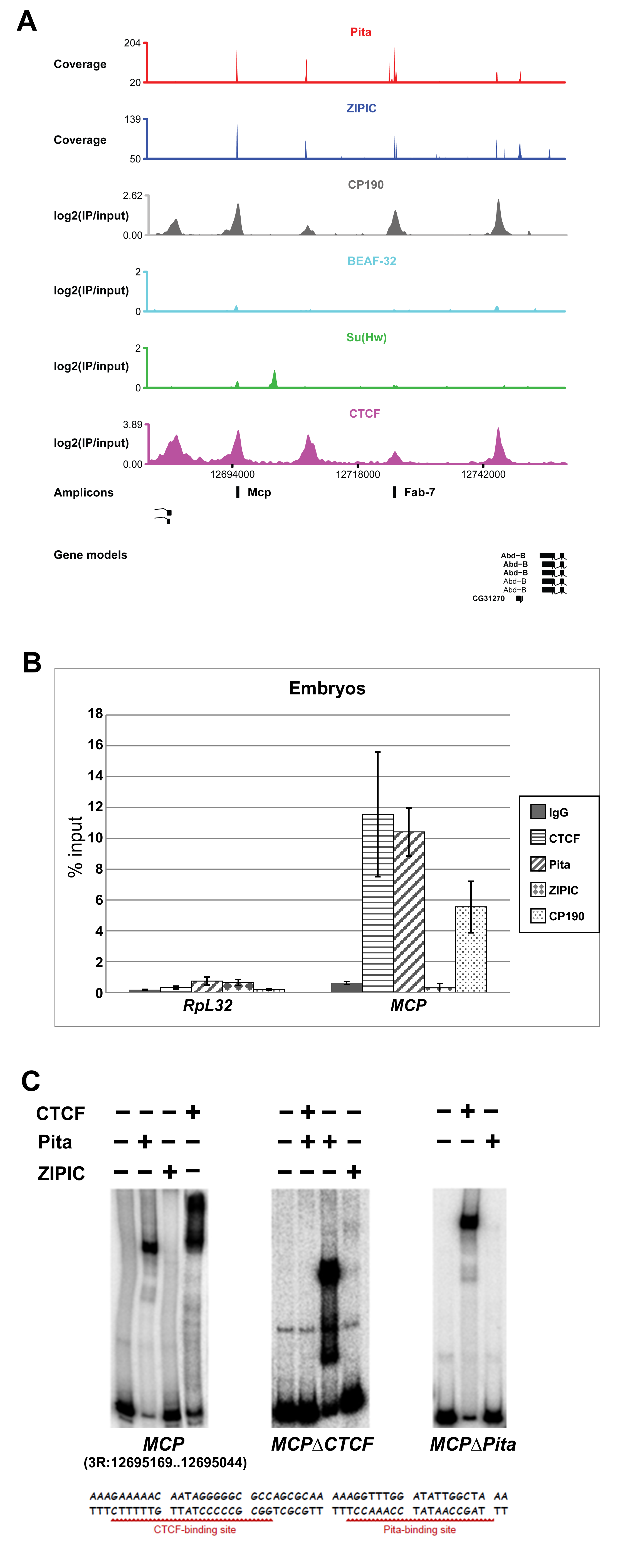

Supplement: Supplemental Material [file supp_gr.174169.114_Fig_S12.tif]
