# Supplementary material for: Two new insulator proteins, Pita and ZIPIC, target CP190 to chromatin
Source: Genome Res. 2015 Jan;25(1):89–99. doi: 10.1101/gr.174169.114 (PMC4317163; doi:10.1101/gr.174169.114)
Supplement: Supplemental Material [file supp_gr.174169.114_Supplemental_Material.docx]

**Supplemental material**

This supplemental material contains the following items.

1. Supplemental methods

2. Supplemental references

3. Supplemental figure legends

4. Supplemental table

**Supplemental methods**

**S2 cell nuclear lysate preparation and mass spec analysis**

S2 cells grown in SFX medium were collected by centrifugation at 700 x g for 5 min, washed once with 1×PBS and resuspended in IP-0 buffer (10 mM Tris-HCl, pH 8.0; 10 mM NaCl, 10 mM MgCl_2_, 1 mM EDTA, 1 mM EGTA, 250 mM sucrose, 1 mM PMSF, 0.2% NP-40, Calbiochem Complete (EDTA-free) Protease Inhibitor Cocktail V), incubated for 10 minutes at 4°C and disrupted by 20 strokes in a Dounce homogenizer on ice. The nuclei were pelleted at 3000 x *g* for 10 min and resuspended in IP-10+ buffer (10 mM Tris-HCl, pH 8.0; 10 mM NaCl, 10 mM MgCl_2_, 1 mM EDTA, 1 mM EGTA, 10% Glycerol, 0.1% NP-40. An equal volume of IP-850+ (10 mM Tris-HCl, pH 8.0; 850 mM NaCl, 10 mM MgCl_2_, 1 mM EDTA, 1 mM EGTA, 10% glycerol, 0.1% NP-40) was added, and the nuclei were lysed for 10 minutes on ice; then two volumes of IP-10+ with 1 U/mL DNase I were added, and incubation was continued for 10 min at room temperature, with rotation. The lysate was cleared by centrifugation at 16 000 x *g* for 20 min and used in immunoprecipitation assays.

S2 cells were stably transfected with pRm-HA/FLAG-CP190 and the expression of cell clones induced with 500 mM CuSO_4_ for 24 h. Nuclear extract preparation was performed as described ([Yusufzai et al. 2004](#_ENREF_14)) and was done for the induced and the uninduced cell clone separately. After immunoprecipitation with anti-FLAG antibody proteins were eluted from the beads and loaded onto a SDS-PAGE (4-12% Bis-Tris Invitrogen). Gels were stained with colloidal blue (Invitrogen), and evenly sized gel pieces were excised and processed for mass spectrometry. The gel pieces were subjected to in gel reduction and alkylation, followed by trypsin digestion as described previously ([Shevchenko et al. 2006](#_ENREF_11)). Briefly, gel pieces were washed twice with 50% (50 mM NH_4_HCO_3_ eluent additive for LC-MS (Sigma-Aldrich) / 50% ethanol) for 20 min and dehydrated with 100% ethanol for 10 min and then vacuum centrifuged. Gel pieces were reduced with 10 mM DTT for 45 min at 56°C and alkylated with 55 mM iodoacetamide for 30 min at RT in the dark. After two steps of washing / dehydration, samples were dehydrated twice with 100% ethanol for 15 min and vacuum centrifuged. Gel pieces were digested overnight at 37°C in 50 µl of digestion buffer containing 12.5 ng/µl of Sequencing Grade Modified Trypsin (Promega Corp., Madison, USA). Released peptides were extracted (collecting separately the liquid mixture of each samples at each step) once by adding 100 µl of 30% acetonitrile LC/MS grade (Thermo Scientific) / 3% trifluoracetic acid (TFA, protein sequence analysis grade, Sigma-Aldrich) in water (LC/MS grade quality, Thermo Scientific), twice by adding 70% acetonitrile, followed by two final extractions with 100% acetonitrile. Extracts were vacuum centrifuged to remove acetonitrile and subsequently acidified with 0.5% TFA. Samples containing tryptic peptides were desalted and concentrated with homemade "STAGE" tips (Stop and Go extraction tips) filled with C-18 (C18 Empore Disks, 3M, Minneapolis, MN) as described earlier ([Rappsilber et al. 2003](#_ENREF_10); [Kruger et al. 2008](#_ENREF_4)).

Mass spectrometric experiments were performed on a nano-flow HPLC system (Agilent) connected to a LTQ-Orbitrap XL instrument (Thermo Fisher Scientific) equipped with a nano electrospray source (Proxeon). The mass spectrometer was operated in the data dependent mode to monitor MS and MS/MS spectra. Survey full-scan MS spectra (from m/z 300–2000) were acquired in the Orbitrap with a resolution of R=60,000 at m/z 400 after accumulation of 1,000,000 ions. The five most intense ions from the preview survey scan delivered by the Orbitrap were sequenced by collision-induced dissociation (CID) in the LTQ. Mass spectra were analyzed using MaxQuant software (Version 1.1.14.10) ([Cox and Mann 2008](#_ENREF_1)) and all tandem mass spectra were searched against the *Drosophila* International Protein Index protein sequence database (IPI version r5.1) and concatenated with reversed copies of all sequences. The required false positive rate was set to 1% at the protein and peptide level. Maximum allowed mass deviation was set to 7 ppm in MS mode and 0.5 Da for MS/MS peaks. Cysteine carbamido-methylation was searched as a fixed modification and N-acetyl methionine, and phospho (STY) was searched as variable modifications. A maximum of three missed cleavages were allowed. Protein quantitation was performed with the MaxQuant label free option as described in ([Luber et al. 2010](#_ENREF_6)) and the ratio between the extract from induced cells and the extract from uninduced cells was determined. A ratio of higher than 3.5 fold was considered to be specific.

**RNA interference in *Drosophila* S2 cells**

The S2 cells were cultured in SFX medium (HyClone) in 60-mm Petri dishes at 25°C. RNAi experiments were performed when the culture reached a density of ~0.2x10^6^ cells/ml. The cells were treated with 100 μg of corresponding dsRNA, and the treatment was repeated after 3 days. On day 7 after the first dsRNA treatment, the cells were used for subsequent experiments (preparation of total protein, total RNA, and cross-linked chromatin). The sequences of PCR primers used to produce DNA templates for dsRNA synthesis are listed below. dsRNA were synthesized with MEGAScript kit (Pierce) according to manufacturer’s recommendations.

**RNA isolation and real-time PCR**

Total RNA was isolated using the TRI reagent (Molecular Research Center, United States) according to the manufacturer’s instructions. RNA was treated with two units of Turbo DNase I (Ambion) for 30 min at 37°C to eliminate genomic DNA. The synthesis of cDNA was performed using 2 mg of RNA, ArrayScript reverse transcriptase (Ambion), and oligo(dT) as a primer. The amounts of specific cDNA fragments were quantified by real-time PCR. At least three independent measurements were made for each RNA sample. Relative levels of mRNA expression were calculated in the linear amplification range by calibration to a standard genomic DNA curve to account for differences in primer efficiencies. Individual expression values were normalized with reference to *RpL32* and *γTub37C* mRNA.

**Plasmid construction**

For protein purification, protein-coding fragments were PCR-amplified using corresponding primers and subcloned into pGEX-4T1 (GE Healthcare), pMAL-C5X (New England Biolabs), or pET32a(+) vector (Merck Biosciences) in frame with the corresponding tag. In case of 6×His-fusions without Thioredoxin, its coding sequence was excised from pET32a(+) vector with *Nde*I. For co-expression assays, proteins fused with 6×His tag were cloned in vector derived from pACYC and pET28a(+) (Novagen) that contained the p15A replication origin, Kanamycin resistance gene, and pET28a(+) MCS.

For protein expression in S2 cells, protein-coding sequences were cloned in frame with 3×FLAG, excised, and subcloned into the pAc5.1 plasmid (Invitrogen).

Plasmids for yeast two-hybrid assay were prepared with pGBT9 and pGAD424 vectors from Clontech. Pita- and ZIPIC-containig derivatives were fused with GAL4 activating domain, and CP190-containing variants were fused with GAL4 DNA-binding domain.

To generate transgenic flies the corresponding vectors containing defective inverted repeats of P-element were prepared. The 3-kb *Sal*I–*Bam*HI fragment containing the *yellow* regulatory region (yr) with the body and wing enhancers (fragment –2873 to –1266 bp relative to the transcription start site) was subcloned into the pGEM7 plasmid digested with *Bam*HI and *Xho*I. The 660-bp PRE from *Ultrabithorax* gene surrounded by frt sites (for FLP recombinase) was then inserted at –1868 from the *yellow* transcription start site (yr-PRE). The 5-kb *Bam*HI–*Bgl*II fragment containing the *yellow* coding region (yc) was subcloned into CaSpeRΔ700 (yc-C700). The CaSpeRΔ700 vector contains the *mini-white* gene without insulator at its 3’ side and defective inverted repeats of P element. The 5×Pita and 4×ZIPIC binding sites and *MCP*, d100C (wild-type or with a mutated Pita-binding site) were inserted between two lox sites (for CRE recombinase). These fragments were inserted into the yr-PRE plasmid between enhancers and *yellow* promoter with *Eco*47III at position –893 from the *yellow* transcription start site. Finally, this region (including *yellow* enhancers, PRE, *yellow* promoter, and the tested fragment) was associated with yc-C700 digested with *Xba*I and *Bam*HI.

**Antibodies**

Antibodies against ZIPIC [84–257 aa], Pita [99–302 and 550–683 aa], CP190 [308–1096 aa], and dCTCF fragments were raised in rabbits and rats and purified from the sera by ammonium sulfate fractionation followed by affinity purification on CNBr-activated Sepharose (GE Healthcare, United States) according to standard protocols. Anti-FLAG M2 antibody was from Sigma (Unites States), anti-GST antibody was from Pierce (United States) and anti-lamin ADL67.10 antibody was from the Hybridoma Bank at the University of Iowa.

**Immunostaining of polytene chromosomes**

*Drosophila* 3^rd^ instar larvae were cultured at 18°C under standard conditions. Polytene chromosome staining was performed as described ([Murawska and Brehm 2012](#_ENREF_8)). The following primary antibodies were used: anti-CP190 Bx63 (mouse) 1:2, anti-Pita (rat) 1:10 and anti-ZIPIC (rat) 1:10. The applied secondary antibodies were AlexaFluor488 goat anti-mouse 1:200 and AlexaFluor568 goat anti-rat 1:200 (Invitrogen). The polytene chromosomes were co-stained with Hoechst33342 (AppliChem).

Analysis was performed with a Zeiss fluorescence microscope (Axio Observer.Z1) which has integrated an OptiGrid Structured Illumination Microscopy system (Qioptiq). AdobePhotoshop was used to process images.

**Immunoprecipitation of protein nuclear extract**

Rabbit antibodies against CP190 (1:500), Pita (1:100), and ZIPIC (1:100) were conjugated with Protein A agarose beads (Pierce, United States); in respective control experiments, rabbit preimmune serum was used. An aliquot of an antibody was mixed with 30 µL of agarose beads equilibrated in HEMG-100 buffer with 100 mM KCl and 0.1% NP-40 and incubated on a rotator at room temperature for 2 h. The beads were then washed with HEMG-100. The nuclear extract (40 mg protein) was adjusted to a volume of 250 µL with HEMG-100, mixed with antibody-conjugated beads, and incubated on a rotator overnight at 4°C. The beads were then washed with two portions of HEMG-100, three portions of HEMG-500 (with 500 mM KCl), and one portion of HEMG-100, resuspended in SDS-PAGE loading buffer, boiled, and analyzed by Western blotting. Proteins were detected using the SuperSignal West Fempto substrate (Pierce).

**Chromatin immunoprecipitation from S2 cells**

Samples of 10^7^ S2 cells in 10 mL of SFX medium were treated with 37% formaldehyde added to a final concentration of 1% and incubated on a rotator at room temperature for 10 min. Cross-linking was stopped by the addition of 0.125 M glycine, and the samples were washed with three portions of PBS (pH 8), containing0.5 mM PMSF and were pelleted at 1000 rpm, 4°C, for 5 min. The pellet was resuspended in 10 mL of buffer I (25 mM HEPES, pH 7.8; 1.5 mM MgCl_2_, 10 mM KCl, 0.1% NP-40, 1 mM DTT, 0.5 mM PMSF, Calbiochem Cocktail V) and placed on ice for 10 min. The suspension was then homogenized by 20 strokes in a Dounce homogenizer using a B pestle and was then centrifuged at 2000 rpm, 4°C, for 5 min. The pellet was resuspended in 5 mL of buffer II (50 mM HEPES, pH 7.8; 140 mM NaCl, 1 mM EDTA, 1% Triton X-100, 0.1% sodium deoxycholate, 0.1% SDS, 0.5 mM PMSF, Calbiochem Cocktail V), sonicated in a Bioruptor sonifier (Diagenode) for 15 min, with alternating 30-s ON/OFF intervals, and centrifuged at 14,000 rpm, 4°C, for 5 min to remove cell debris. A 50 µL aliquot of the supernatant was used to test the results of sonication and to measure DNA concentration: the sample was diluted with 350 µL of elution buffer (50 mM Tris, pH 8.0; 1 mM EDTA, 1% SDS, 50 mM NaHCO_3_) and treated with RNase A (1 µL from 10 mg/mL stock) at 37°C for 1 h and then with proteinase K (1 µL from 20 mg/mL stock) at 42°C for 2 h. After subsequent incubation at 65°C for 6 h, DNA was isolated by phenol–chloroform–isoamyl alcohol extraction, concentrated by ethanol precipitation with glycogen (each tube was supplemented with 5 µL of glycogen (from 20 mg/mL stock), 40 µL of sodium acetate (from 3 M stock), and 1 mL of ethanol, vortexed, and placed at –20°C for 4–5 h). The precipitated DNA was pelleted at 14,000 rpm for 30 min, washed with 80% ethanol, and resuspended in 50 µL of MilliQ water. Protein A Sepharose (Pierce) was washed with three portions of buffer II and incubated with 1 mg/mL BSA in the same buffer on a rotator at 4°C for 4 h. Chromatin samples containing 10–20 µg of DNA equivalent were each diluted with buffer II to a final volume of 1 mL, their 50 µL aliquots were stored as input, and then the samples were incubated overnight, at 4°C, with appropriate antibodies (rabbit antibodies against Pita (1:1000), ZIPIC (1:200), or CP190 (1:500), or with nonspecific IgG purified from rabbit preimmune sera (control)) in the presence of blocked Protein A Sepharose beads (40 µL). After incubation, the beads were washed on a rotator, at 4°C, with three 1 mL portions of buffer II and two 1 mL portions of buffer III (50 mM HEPES, pH 7.8; 500 mM NaCl, 1 mM EDTA, 1% Triton X-100, 0.1% sodium deoxycholate, 0.1 % SDS, 0.5 mM PMSF, Calbiochem Cocktail V), each wash for 10 min. Then 400 µL of elution buffer was added, and the beads were treated with RNase A (1 µL from 10 mg/mL stock) at 37°C for 1 h and, after adding 20 µL of NaCl (from 4 M stock), with proteinase K (1 µL from 20 mg/mL stock) at 42°C for 2 h. The samples were incubated on a thermoshaker at 65°C overnight, and DNA was isolated by phenol–chloroform–isoamyl alcohol and chloroform–isoamyl alcohol extraction in phase-lock tubes. The samples were concentrated by ethanol precipitation with glycogen (as described above), and resuspended in MilliQ water.

**Chromatin immunoprecipitation from pupae and embryos**

Chromatin was prepared from mid-late pupae or 0- to 12-h embryos. A 500-mg sample was ground in a mortar in liquid nitrogen and resuspended in 10 mL of buffer A (15 mM HEPES-KOH, pH 7.6; 60 mM KCl, 15 mM NaCl, 13 mM EDTA, 0.1 mM EGTA, 0.15 mM spermine, 0.5 mM spermidine, 0.5% NP-40, 0.5 mM DTT) supplemented with 0.5 mM PMSF and Calbiochem Cocktail V). The suspension was then homogenized in a Dounce homogenizer with pestle B and filtered through Nylon Cell Strainer (BD Biosciences, United States). The homogenate was transferred to 3 mL of buffer A with 10% sucrose (AS), and the nuclei were pelleted by centrifugation at 4 000 *g*, 4°C, for 5 min. The pellet was resuspended in 5 mL of buffer A, homogenized again in a Dounce homogenizer, and transferred to 1.5 mL of buffer AS to collect the nuclei by centrifugation. The nuclear pellet was resuspended in wash buffer (15 mM HEPES-KOH, pH 7.6; 60 mM KCl, 15 mM NaCl, 1 mM EDTA, 0.1 mM EGTA, 0.1% NP-40, protease inhibitors) and cross-linked with 1% formaldehyde for 15 min at room temperature. Cross-linking was stopped by adding glycine to a final concentration of 125 mM. The nuclei were washed with three 10-mL portions of wash buffer and resuspended in 1.5 mL of nuclear lysis buffer (15 mM HEPES, pH 7.6; 140 mM NaCl, 1 mM EDTA, 0.1 mM EGTA, 1% Triton X-100, 0.5 mM DTT, 0.1% sodium deoxycholate, 0.1% SDS, protease inhibitors). The suspension was sonicated in a Bioruptor sonifier (15 alternating 30-s ON and 60-s off intervals), and its 50-µL aliquot was used to test the results of sonication and measure DNA concentration. Debris was removed by centrifugation at 14 000 *g*, 4°C, for 10 min, and chromatin was pre-cleared with Protein A agarose (Pierce) blocked with BSA and salmon sperm DNA, with 50 µL aliquots of such pre-cleared chromatin being stored as input. Samples containing 10–20 µg of DNA equivalent in 1 mL of nuclear lysis buffer were incubated overnight, at 4°C, with rabbit antibodies against Pita (1:1000), ZIPIC (1:200), dCTCF (1:1000), or CP190 (1:500), or with nonspecific IgG purified from rabbit preimmune sera (control). Chromatin–antibody complexes were collected using blocked Protein A agarose at 4°C over 5 h. After several rounds of washing with lysis buffer (as such and with 500 mM NaCl), LiCl buffer (20 mM Tris-HCl, pH 8; 250 mM LiCl, 1 mM EDTA, 0.5% NP-40, 0.5% sodium deoxycholate, protease inhibitors), and TE buffer (10 mM Tris-HCl, pH 8; 1 mM EDTA), the DNA was eluted with elution buffer (50 mM Tris-HCl, pH 8.0; 1 mM EDTA, 1% SDS), the cross-links were reversed, and the precipitated DNA was extracted by the phenol–chloroform method. The enrichment of specific DNA fragments was analyzed by real-time PCR, using a StepOne Plus Thermal Cycler (Applied Biosystems, United States). The primers used for PCR in ChIP experiments for genome fragments are shown below.

**Deep sequencing of ChIP DNA**

Samples were prepared as described for ChIP, but the elution step after DNA purification was performed with H_2_O instead of elution buffer. If necessary samples generated with the same antibody were pooled and volume was reduced by evaporation to 30 μl to obtain at least 10 ng of total DNA. Sequencing libraries were prepared from 10 ng of immunoprecipitated DNA with the Illumina ChIPSeq DNA Sample Prep Kit according to Illuminas’ instructions. Cluster generation was performed using the Illumina cluster station, sequencing on the Genome Analyzer IIx followed a standard protocol. The fluorescent images were processed to sequences using the Genome Analyzer Pipeline Analysis software 1.8 (Illumina). We sequenced an input sample as well as one sample for Pita and ZIPIC chromatin-immunoprecipitates, respectively. Individual ChiP-seq data were validated extensively by specific qPCR. Raw and processed data have been deposited in the NCBI gene expression omnibus (GEO) under accession number GSE54337.

Read statistics:

|  | No. of reads | mapable reads | unique |
| --- | --- | --- | --- |
| ZIPIC | 38691262 | 25258265 | 18687831 |
| Pita | 43124394 | 10293386 | 7618590 |
| Input | 46465514 | 29723121 | 25907814 |

**ChIP-sequencing analyses and peak calling**

Processing of ChIP-seq reads ChIP-Seq reads were converted to fastq format and aligned to a precompiled dm3 reference index with BOWTIE ([Langmead et al. 2009](#_ENREF_5)). Sequencing data were controlled for general quality features using FastQC. Unambiguously mapped and unique reads were kept for subsequent generation of binding profiles and calling of peaks using MACS ([Zhang et al. 2008](#_ENREF_15)) and PeakRanger ([Feng et al. 2011](#_ENREF_2)) using reads derived from sequencing of input DNA as control. Peaks were called at p < 10^-5^ and FDR < 5%. All downstream analyses were done in R/BioConductor (http://www.bioconductor.org). Two peaks were determined to be overlapping in case they had a minimal overlapping interval of 1bp.

**Visualization of binding profiles**

After extension of reads, continuous coverage vectors were calculated and normalized per million reads to account for differential library sizes. These data were used to collect data in windows of different sizes spanning features of interest (e.g. transcription factor peaks) using the sitepro ([Shin et al. 2009](#_ENREF_12)) application. The binding data was binned across binding sites in 50 bp windows and the mean was calculated at each position in order to generate cumulative binding profiles.

**Motif search and analysis**

We extracted the sequences in a ± 50bp window around the peak maxima from top 500 peaks. These sequences were used to run MEME-ChIP (http://meme.ebi.edu.au/) ([Machanick and Bailey 2011](#_ENREF_7)) in order to identify *de novo* motifs and known motifs enriched within peak regions of respective factors. In order to study the relationship between occurrences of motifs and binding strength of corresponding factors we used a PWM representation of the motifs identified as mentioned above. We used the matchPWM function of the Biostrings package within R/BioConductor to rank all binding sites with respect to motif quality of the best motif identified in a given interval.

**Comparison to published data sets**

In order to compare ChIP-seq binding data with gene expression we took Affymetrix microarray data to get gene expression estimates for all *Drosophila* genes as expressed in S2 cells ([Page et al. 2005](#_ENREF_9)). For comparison with other transcription factor binding data we downloaded processed binding data from ChIP-chip experiments conducted by the modENCODE Consortium ([The modENCODE Consortium et al. 2010](#_ENREF_13)). In order to enable comparison to our ChIP-seq data, all data was binned into 100 bp bins and transformed by Z-normalization. For correlation analysis we either calculated Pearson's R over all bins genome-wide or all bins associated with regions of significant binding for a pair of two given binding factors. Alternatively we downloaded the peak intervals as detected by the modENCODE Consortium and determined the overlap between all available peak sets and Pita or ZIPIC. Two peaks were called overlapping in case they had a minimal overlapping interval of 1bp. For a given comparison between two peak sets the expected overlap was calculated based on a random distribution of binding peaks. The ratio between observed and expected overlap was used as a score to rank the degree of overlap determined for multiple comparisons.

**Comparison to genomic annotations**

RefSeq gene annotations for *Drosophila melanogaster* were downloaded from UCSC homepage (version dm3). Next the genome was partitioned into the following intervals: transcriptional start site (TSS; +/- 1 kb around RefSeq start sites), TSS upstream (-10 kb to -1 kb), transcriptional end sites (TES; +/- 1 kb around transcriptional end sites), exons, introns and everything not covered by these classes as intergenic. ZIPIC, Pita and CP190 peak ranges were intersected with these annotation intervals and the relative association was calculated (as fraction of the complete genome) and compared to the genomic background distribution.

**Electrophoretic mobility shift assay (EMSA)**

Aliquots of purified recombinant proteins (10–15 μg) were incubated with a radioactively labeled DNA fragments (artificial Pita- and ZIPIC-binding sites, *MCP*, 57B5, 60A9L, 60A9R, 66E5, 67B6, 100B7, 100C regions; 100 cps) in the presence of nonspecific binding competitor poly(dI-dC). Incubation was performed in PBS (pH 8) containing 5 mM MgCl_2_, 0.1 mM ZnSO_4_, 1 mM DTT, 0.1% NP-40, and 10% glycerol, at room temperature for 30 min. The mixtures were then resolved by nondenaturing 5% PAGE in 0.5× TBE buffer at 5 V/cm.

**Generation and analysis of transgenic lines**

The transgenic construct and P25.7wc plasmid were injected into *yacw^1118^* preblastoderm embryos ([Karess and Rubin 1984](#_ENREF_3)). The resultant flies were crossed with *yacw^1118^* flies, and the transgenic progeny were identified by their eye color under a Stemi 2000 stereomicroscope (Carl Zeiss, Germany).The transformed lines were tested for transposon integrity and copy number by RT-PCR. Only single-copy transformants were included in the study.

The lines with DNA fragment excisions were obtained by crossing the transposon-bearing flies with the Flp (*w^1118^; S2CyO, hsFLP, ISA/Sco ;+*) or Cre (*yw; Cyo, P[w+,cre]/Sco; +*) recombinase-expressing lines*.* The Cre recombinase induces 100% excisions in the next generation. The high level of Flp recombinase was produced by heat shock treatment for 2 h during the first 3 days after hatching. All excisions were confirmed by PCR analysis. Details of the crosses and primers used for genetic analysis and the excision of functional elements are available upon request.

To determine the levels of *yellow* expression, we visually estimated the degree of pigmentation in the bristles, abdominal cuticle, and wing blades of 3- to 5-day-old males developing at 25°C. At least 50 flies were scored independently by two people for each *y* line. A five-grade scale was used, with grade 1 corresponding to the total loss of *yellow* expression and grade 5 corresponding to wild-type pigmentation. The degree of *yellow* expression in bristles of the thorax and head was scored using a 5-point scale, where 1 denotes loss of pigmentation in all bristles on the thorax and head; ev, extreme variegation (only 1–3 bristles on the thorax and head are partially pigmented); mv, moderate variegation (about half of bristles are yellow); wv, weak variegation (only 1–3 bristles on the thorax and head are yellow or partially pigmented); and 5, pigmentation of all bristles as in wild-type flies.

**List of primers.**

***Oligos used for the synthesis of binding sites***

Pita_bs_d 5’-actttagccaagacgcg-3’

Pita_bs_r 5’-attagatcttcggattcgggttcgcgtcttggctaaagtttcggattcgggttcgcgtcttggctaaagt-3’

ZIPIC_bs_d 5’-cagctgagcgccagg-3’

ZIPIC_bs_r 5’-attagatctgctgcagtttgcagccctggcgctcagctgtgctgcagtttgcagccctggcgctcagctg-3’

***Oligos used for RNA interference***

ZIPIC_i1_d 5’-ctaatacgactcactatagggagaTTTGCTGCATTTGTCAATTCTCG-3’

ZIPIC_i1_r 5’-ctaatacgactcactatagggagaTTCTGCTTGTGGTCCTTGGACAT-3’

ZIPIC_i2_d 5’-ctaatacgactcactataGCCGAAGAGGAGCACAC-3’
ZIPIC_i2_r 5’-ctaatacgactcactataGGCAGGCGAACTGCTTC-3’

Pita_i_d 5’-ctaatacgactcactatagggagaTCAGTATCCGCTCACGGGTAACTG-3’

Pita_i_r 5’-taatacgactcactatagggagaCTGCGCGAGCGAGTGTGATTCAGCCGATGGAT-3’

Cp190_i_d 5’-ctaatacgactcactatagggagaTACGACAACAAGCAGCAGTGCAT-3’

Cp190_i_r 5’-ctaatacgactcactatagggagaTATCATATTCCTCTTCGGTGCCG-3’
GFP_i _ d 5’-ctaatacgactcactatagggaGATGCGACGTAAACGGCCACAAGTTC-3’

GFP_i _ r 5’-ctaatacgactcactatagggaGATTCCAGCTTGTGCCCCAGGATGTT-3’

***Primers used for amplification of fragments for EMSA***

*MCP*_d 5’-AAACTTAACTCAGACTTGG-3'

*MCP*_r 5’-CCCAATCGTTGTAAGTGT-3'

100C_d 5’- TGCCAAACCTACCTACACATAAAT-3'

100C_r  5’-CCCGAGGTTTCAACTTTCATA -3'

60A9L_d   5’-ATTTCATGACAGGCTGTGG-3'

60A9L_r   5’-GCAGGTTAACTAGCTATGCG-3'

100B7_d 5’-GTGTGTGCGAGTGTGCCG-3'

100B7_r 5’-GCAGTTTCGTGGGAAAGGG-3'

57B5_d     5’-ATGTGGATAAACGTGCACACCACC-3'

57B5_r     5’-TGTCATTCAGTGTAGGGTTGCCAG-3'

60A9R_ d   5’-GTGTTTAACTTAGTTGTGCCATC-3'

60A9R_ r   5’-ACCTTTACCAATCGCATTCA-3'

66E5_d    5’-CATGGCACTGCCGTTCAACCAAAT-3'

66E5_r    5’-CAGCAGCAGGTTATGTGAGCAGTT-3'

67B6_d 5’-CACTATGGCACAAATCTAACC-3'

67B6_r 5’-TTTTGGACTCTTACAATACTTT-3'

***Primers used for analysis of ChIP by RT-PCR***

*RpL32*_d 5'-GTTCGATCCGTAACCGATGT-3'
*RpL32*_r 5'-CCAGTCGGATCGATATGCTAA-3'

*γTub37C*_d 5’-GCTTTCCCAAGAAGCTCATACA-3'

*γTub37C*_r 5’-GGTTCAGTGCGGTATTATCCAG-3'

62D_d 5'-TGATACCAGGCGAACAGAAATC-3'

62D_r 5'-TTTGGGCTTGGTGAGAACAG-3'

*Fab-8*_d 5'-TGTTGGTGAGCAAGCGAAGA-3'
*Fab-8*_r 5'-CGAACATTTTTTACGCGACATGT-3'

*Fab-7*_d 5'-TAAGCCAACTGGTTTCCAACTCT-3'
*Fab-7*_r 5'-TTGCCCAGGGTAAGTAACGGTAT-3'

C1_d 5'-AACCACTTTATCTGCGGAGGTCGT-3'

C1_r 5'-GCCCAAATGCACCTGCTTGCTAAA-3'

C2(5xP_bs)_d 5'-ACCCGAATCCGAAGATCTAA-3'

C2(*MCP*)_d 5'-GCGTCGCTTATGTACTCTG-3'

C2(100C)_d 5’-TATGAAAGTTGAAACCTCGGG-3’

C2_r 5'-TGGTTTCCGCTAGTTATTGG-3'

*MCP*_c_d 5’-CGCGGCCATGTATTATGT-3'

*MCP*_c_r 5’-TACAACGCTTGGGTTTCTC-3'

100C_c_d 5’-CGACAGCATGTAACAGGTATAA-3'  
100C_ r 5’-CCCGAGGTTTCAACTTTCATA-3'

60A9L_c_d 5’-AGACGCAAACCCTCCCAG-3'  
60A9L_c_r 5’-TGGTGCAATAAAGGTGAGGC-3'

100B7_c_d   5’-CAAGGGCAACCTCCTATTC-3'

100B7_c_r   5’-ACTGGAAAGTACCAGAAATACC-3'

57B5_d     5’-ATGTGGATAAACGTGCACACCACC-3'

57B5_r     5’-TGTCATTCAGTGTAGGGTTGCCAG-3'

60A9R_ d   5’-GTGTTTAACTTAGTTGTGCCATC-3'

60A9R_ r  5’-ACCTTTACCAATCGCATTCA-3'

66E5_d    5’-CATGGCACTGCCGTTCAACCAAAT-3'

66E5_r    5’-CAGCAGCAGGTTATGTGAGCAGTT-3'

67B6_c_d    5’-TTTACAGAGCAGGGTTGTCAGGGT-3'

67B6_c_r     5’-CTGCGACTTTGAGCGATACTTTCAC-3'

***Primers used for analysis of gene expression level by RT-PCR***

*RpL32*_d 5'-GTTCGATCCGTAACCGATGT-3'
*RpL32*_r 5'-CCAGTCGGATCGATATGCTAA-3'

*γTub37C*_d 5’-GCTTTCCCAAGAAGCTCATACA-3'

*γTub37C*_r 5’-GGTTCAGTGCGGTATTATCCAG-3'

*Pita*_d 5'-ATGCTGACCGAGAAGAGGGTCT-3' 
*Pita*_r 5'-GCCATAATCTGCAGCGGCAGATTGG-3'

*ZIPIC*_d   5'-AGAAGCTCGCCAGATACCACAAGT-3' 
*ZIPIC*_r   5'-TCCTGTTGCTGTGCCTCCTCAATA-3'

*CP190*_d 5'-TAACAAGAGACCAGCACAGACAA-3'

*CP190*_r 5'-TAAAGCATTCCTCCATCGTACTC-3'

**Supplemental References**

Cox J, Mann M. 2008. MaxQuant enables high peptide identification rates, individualized p.p.b.-range mass accuracies and proteome-wide protein quantification. *Nat Biotechnol* **26**(12): 1367-1372.

Feng X, Grossman R, Stein L. 2011. PeakRanger: a cloud-enabled peak caller for ChIP-seq data. *BMC Bioinformatics* **12**: 139.

Karess RE, Rubin GM. 1984. Analysis of P transposable element functions in Drosophila. *Cell* **38**(1): 135-146.

Kruger M, Moser M, Ussar S, Thievessen I, Luber CA, Forner F, Schmidt S, Zanivan S, Fassler R, Mann M. 2008. SILAC mouse for quantitative proteomics uncovers kindlin-3 as an essential factor for red blood cell function. *Cell* **134**(2): 353-364.

Langmead B, Trapnell C, Pop M, Salzberg SL. 2009. Ultrafast and memory-efficient alignment of short DNA sequences to the human genome. *Genome Biol* **10**(3): R25.

Luber CA, Cox J, Lauterbach H, Fancke B, Selbach M, Tschopp J, Akira S, Wiegand M, Hochrein H, O'Keeffe M et al. 2010. Quantitative proteomics reveals subset-specific viral recognition in dendritic cells. *Immunity* **32**(2): 279-289.

Machanick P, Bailey TL. 2011. MEME-ChIP: motif analysis of large DNA datasets. *Bioinformatics* **27**(12): 1696-1697.

The modENCODE Consortium, Roy S, Ernst J, Kharchenko PV, Kheradpour P, Negre N, Eaton ML, Landolin JM, Bristow CA, Ma L et al. 2010. Identification of functional elements and regulatory circuits by Drosophila modENCODE. In *Science*, Vol 330, pp. 1787-1797.

Murawska M, Brehm A. 2012. Immunostaining of Drosophila polytene chromosomes to investigate recruitment of chromatin-binding proteins. *Methods in molecular biology* **809**: 267-277.

Page AR, Kovacs A, Deak P, Torok T, Kiss I, Dario P, Bastos C, Batista P, Gomes R, Ohkura H et al. 2005. Spotted-dick, a zinc-finger protein of Drosophila required for expression of Orc4 and S phase. *EMBO J* **24**(24): 4304-4315.

Rappsilber J, Friesen WJ, Paushkin S, Dreyfuss G, Mann M. 2003. Detection of arginine dimethylated peptides by parallel precursor ion scanning mass spectrometry in positive ion mode. *Analytical chemistry* **75**(13): 3107-3114.

Shevchenko A, Tomas H, Havlis J, Olsen JV, Mann M. 2006. In-gel digestion for mass spectrometric characterization of proteins and proteomes. *Nat Protoc* **1**(6): 2856-2860.

Shin H, Liu T, Manrai AK, Liu XS. 2009. CEAS: cis-regulatory element annotation system. *Bioinformatics* **25**(19): 2605-2606.

Yusufzai TM, Tagami H, Nakatani Y, Felsenfeld G. 2004. CTCF tethers an insulator to subnuclear sites, suggesting shared insulator mechanisms across species. *Molecular cell* **13**(2): 291-298.

Zhang Y, Liu T, Meyer CA, Eeckhoute J, Johnson DS, Bernstein BE, Nusbaum C, Myers RM, Brown M, Li W et al. 2008. Model-based analysis of ChIP-Seq (MACS). *Genome Biol* **9**(9): R137.

**Supplemental Figure Legends**

**Figure S1. Testing interactions of CP190 with Pita or ZIPIC** ***in vivo***

(A) Western blots of proteins from S2 cells treated with Pita- or ZIPIC -specific and mock dsRNAs show knockdown of the corresponding proteins. The antibodies used for detection are indicated on the right of each blot. (B) Nuclear extracts from *Drosophila* embryos were immunoprecipitated with antibodies against Pita, or ZIPIC (using nonspecific IgG as a negative control), and the immunoprecipitates (IP) were analyzed by western blotting for the presence of Pita and ZIPIC. Inputs show the starting samples of nuclear extract, outputs are supernatant after sedimentation of immunoprecipitated material. (C) Coimmunolocalization of CP190 (green) and Pita or ZIPIC (red) proteins on polytene chromosomes. DNA is stained blue.

**Figure S2. Identification of individual Pita and ZIPIC** **domains interacting with CP190 domains in yeast two-hybrid assay.**

For designations, see Fig.2.

**Figure S3. Coverage vectors for ZIPIC (red) and Pita (blue) for an 8-kb region on chromosome 3.**

ChIP-chip track for a CP190 binding profile (downloaded from modEncode ([The modENCODE Consortium et al. 2010](#_ENREF_13))) is shown below.

**Figure S4. Landscape at the selected Pita- and ZIPIC-binding sites.**

Tracks for Pita, ZIPIC, CP190, BEAF32, Su(Hw), dCTCF binding profiles. Locations of amplicons relative to flanking genes are indicated.

**Figure S5. Colocalization of CP190 with Pita and ZIPIC** **on selected chromatin regions in S2 cells.**

(A, B) Bar charts show the results of ChIP for the relative enrichments of Pita or ZIPIC and CP190 in (A) Pita and (B) ZIPIC binding regions on chromatin isolated from S2 cells treated with specific dsRNA (Ri) against *pita*, *ZIPIC* or *CP190* coding regions and incubated with antibodies against (A) Pita or (B) ZIPIC and CP190. C is the mock-treated S2 cells (non-specific eGFP dsRNA). The results are presented as a percentage of input DNA. Relative locations of primers for ChIP on the cytological map are indicated below. Error bars show standard deviations of triplicate PCR measurements. Below are the results of electrophoretic mobility shift assay of recombinant Pita and ZIPIC proteins incubated with corresponding radioactively labeled DNA fragments, which confirm the binding of Pita and ZIPIC to the tested regions. *RpL32* and *γTub37C* points were used as negative controls for binding of Pita, ZIPIC and CP190. At the same time *Fab-8* (dCTCF-dependent) and *62D* (Su(Hw)-dependent) were used as positive controls for binding of CP190. (C) Depletion levels of Pita, ZIPIC and CP190 according to quantitative real-time PCR with cDNAs synthesized on RNAs extracted from S2 cells after treatment with dsRNAs. Individual transcript levels determined by quantitative PCR with corresponding primers were normalized relative to *RpL32* and *γTub37C* for the amount of input cDNA. Error bars show standard deviations of triplicate PCR measurements.

**Figure S6. Combinatorial binding of insulator factors.**

Binary heat maps of all Pita, ZIPIC, CP190, BEAF, dCTCF and Su(Hw) binding sites. Sites have been classified based on the combinatorial co-occurrence of individual factors. Black boxes indicate a given factor to bind to a given category, respectively. The plot is sorted for the number of occurrences, which is a given at the right side of the matrix.

The yellow to blue squares at the left of the plot indicate the relative enrichment/ depletion of a given combination in comparison to simulated binding data. The dendrogram at the top of the figure indicating the degree of similarity between individual factors has been calculated based the binary raw data underlying this figure, consisting of a table all individual binding sites.

**Figure S7. Strong overlap of Pita and ZIPIC with other insulator binding factors.**

Pita and ZIPIC peaks were compared to the peak sets of all ChIP-chip data from the modEncode Consortium ([The modENCODE Consortium et al. 2010](#_ENREF_13)). For each individual comparison the number of overlapping peaks and the expected number of overlaps were calculated. The corresponding lists were sorted for the ratio between observed and expected overlap and the top 20 results are shown for Pita (A) and ZIPIC (B) in decreasing order.

**Figure S8. Correlation analysis reveals similarities between Pita/ZIPIC and CP190 and other insulator factor binding profiles.**

Average coverage (Pita and ZIPIC) or average enrichment of ChIP over input (for modENCODE ChIP-chip data) were calculated across all Pita and ZIPIC peaks, respectively. Pearson´s correlation coefficients were calculated to compare similarity between Pita/ZIPIC binding and modENCODE factors. Data was sorted in decreasing fashion according to the calculated coefficients (A: Pita; B: ZIPIC). Of the 337 analyzed modENCODE data sets ([The modENCODE Consortium et al. 2010](#_ENREF_13)) CP190 and related factors are strongly enriched amongst the profiles with the highest similarity to Pita and ZIPIC.

**Figure S9. *In vitro* binding of Pita and ZIPIC is specific for each factor.**

Electrophoretic mobility shift assay for the interaction of recombinant (A) Pita and (B) ZIPIC with a DNA fragment containing five Pita or four ZIPIC binding sites used in the transgenic construct. (C) Distribution of Pita, ZIPIC, CP190, BEAF-32, Su(Hw), dCTCF binding profiles at the *yellow* regulatory region that was used for ChIP analysis of transgenic constructs.

**Figure S10. Five classes of abdominal pigmentation.**

The five-grade scale of the *yellow* pigmentation level in the abdominal cuticle (reflecting the activity of the body enhancer), with wild-type expression and the absence of expression assigned scores 5 and 1, respectively.

**Figure S11. Testing the enhancer-blocking activity of the Pita-binding site at the 100C region.**

(A) Reductive scheme of transgenic construct used to examine the enhancer-blocking activity of Pita-binding site at the 100C region. This element is shown as a grey box. (B), (C) Panel shows the numbers of transgenic lines with different levels of pigmentation in the abdominal structures. Histograms show binding of Pita and CP190 proteins to d100C region with (B) wild-type or (C) mutated Pita binding site in the transgenic construct. For other designations, see Figure 6.

**Figure S12. Binding of Pita to the *MCP* element and its role in boundary activity.**

(A) Distribution of Pita, ZIPIC, CP190, BEAF-32, Su(Hw), dCTCF binding profiles at the *MCP* region. (B) Results of immunoprecipitation of chromatin isolated from *Drosophila* embryos with antibodies to dCTCF, Pita, ZIPIC and CP190 proteins at the *RpL32* (negative control) and *MCP* regions. (C) Electrophoretic mobility shift assay for the interaction of recombinant dCTCF, Pita, and ZIPIC with the DNA fragment containing a wild-type *MCP* element or the same element with mutated dCTCF or Pita binding sites.

**Table S1**. List of identified CP190-interacting proteins ranked by the ratio of summed peptide intensities derived from material purified from the pRm-HA/FLAG-CP190 expressing S2 cell clone induced with 500 mM CuSO_4_ and from the non-induced cell clone. Shown are the known insulator factors including Pita and ZIPIC.

| Rank | Protein names | Gene  symbol | Unique peptides | Sequence coverage [%] | Ratio of normalized intensities |
| --- | --- | --- | --- | --- | --- |
| 1 | CP190 | *Cp190* | 82 | 82.8 | 26.7 |
| 2 | CP60 | *Map60* | 26 | 57.0 | 19.7 |
| 5 | CTCF | *CTCF* | 11 | 15.2 | 12.0 |
| 6 | Mod(mdg4) | *mod(mdg4)* | 8 | 45.9 | 11.5 |
| 10 | Ibf1 | *Ibf1* | 10 | 54.5 | 10.3 |
| 22 | Pita | *pita* | 24 | 43.0 | 6.4 |
| 28 | Ibf2 | *Ibf2* | 7 | 41.5 | 5.7 |
| 30 | Su(Hw) | *su(Hw)* | 35 | 37.1 | 5.7 |
| 51 | Chromator | *Chro* | 29 | 36.4 | 3.8 |
| 54 | Putzig | *pzg* | 39 | 50.9 | 3.5 |
| 55 | ZIPIC | *CG7928* | 8 | 19.0 | 3.5 |
